# Construction and Characterization of Rabbit scFv Antibodies Targeting Fibronectin by Phage Display Technology

**DOI:** 10.3390/ijms27209074

**Published:** 2026-10-13

**Authors:** Yu-Wei Chiang, Jia-Lin Juang, Yi-Yuan Yang, Sy-Jye Leu, Jun-Jen Liu, Bor-Yu Tsai, Yan-Chiao Mao, Fang-Yi Yen, Jia-Yun Chiang, Valencia Andriani, Priskila Cherisca Thenaka, Ta-Lun Tan, Ting-Jing Shen, Chi-Hsin Lee

**Affiliations:** 1 Graduate Institute of Biodefense, National Defense Medical University, Taipei 114201, Taiwan; 2 Institute of Preventive Medicine, National Defense Medical University, Taipei 114201, Taiwan; 3 Department of Occupational Medicine and Clinical Toxicology, Taipei Veterans General Hospital, Taipei 112201, Taiwan; 4 Department of Biology and Anatomy, National Defense Medical University, Taipei 114201, Taiwan; 5 Zinexts Life Science Corp., New Taipei City 221416, Taiwan; 6 JW Diagnostics Inc., New Taipei City 235048, Taiwan; 7 Department of New Drug Development, GlycoNex Inc., New Taipei City 221021, Taiwan; 8 Department of R&D, Zinexts Life Science Corp., New Taipei City 221416, Taiwan; 9 School of Medical Laboratory Science and Biotechnology, College of Medical Science and Technology, Taipei Medical University, New Taipei City 235603, Taiwan; 10 Ph.D. Program in Medical Biotechnology, College of Medical Science and Technology, Taipei Medical University, New Taipei City 235603, Taiwan; 11 Navi Bio-Therapeutics Inc., Taipei 10351, Taiwan; 12 Division of Clinical Toxicology, Department of Emergency Medicine, Taichung Veterans General Hospital, Taichung 407219, Taiwan; 13 Department of Biotechnology and Laboratory Science in Medicine, National Yang Ming Chiao Tung University, Taipei 112304, Taiwan; 14 iReal Biotechnology Inc., Research and Development Center, New Taipei City 248022, Taiwan; 15 Department of Project Management, GlycoNex Inc., New Taipei City 221021, Taiwan

**Keywords:** fibronectin (FN), rabbit antibody, phage display, single-chain variable fragment (scFv)

## Abstract

Fibronectin (FN) is a multifunctional glycoprotein involved in extracellular matrix remodeling, tumor progression, tissue repair, and host–pathogen interactions. Despite its biological and clinical relevance, FN-binding antibodies derived from non-rodent species remain comparatively less explored. Here, one rabbit was immunized with full-length recombinant human FN, eliciting a robust antibody response confirmed by ELISA and Western blotting. Two rabbit-derived single-chain variable fragment (scFv) phage-display libraries were constructed from peripheral blood mononuclear cells, yielding 5.5 × 10^7^ and 5.0 × 10^7^ transformants for the short- and long-linker formats, respectively. After four rounds of biopanning, phage ELISA confirmed enrichment, and sequence analysis grouped selected clones into five short-linker and four long-linker groups. Seven purified scFvs displayed distinct ELISA binding profiles. Fiber-optic particle plasmon resonance (FOPPR)-based kinetic analysis yielded apparent equilibrium dissociation constants (*K_D,app_*) of 11.21–432.06 nM, with FN-L13 showing the lowest *K_D,app_*. Analytical size-exclusion chromatography indicated predominantly monomeric scFvs. Five scFvs recognized a high-molecular-weight band consistent with fibronectin in mouse plasma by Western blotting. ELISA showed preferential FN binding, with generally low reactivity toward human serum albumin (HSA) and collagens I, III, and IV, although FN-S1 and FN-L1 showed detectable fibrinogen reactivity. These findings support rabbit-derived FN-binding scFvs as research reagents for future FN-related assay development and mechanistic studies.

## 1. Introduction

Tissue architecture and extracellular matrix (ECM) organization are essential for maintaining organ homeostasis and function. The ECM is a dynamic and tissue-specific network of proteins and glycans that provides structural and mechanical support while actively regulating cellular behavior. Its assembly, organization, and remodeling influence a wide range of cellular processes, including adhesion, proliferation, differentiation, migration, and survival [1]. Dysregulation of ECM composition or turnover can profoundly alter tissue architecture and signaling and has been implicated in pathological processes such as fibrosis, inflammation, and cancer progression [2,3,4,5]. Fibronectin (FN) is a large multifunctional glycoprotein and a major component of the ECM that contributes to cell adhesion, migration, proliferation, tissue repair, angiogenesis and cancer metastasis [3]. Alternative splicing of the *FN1* gene generates multiple FN isoforms, including variants containing the extra domains A (EDA) and B (EDB), whose expression is tightly regulated during development and tissue remodeling but is frequently increased under pathological conditions such as inflammation, fibrosis, and tumor progression [3,6]. Accumulating evidence further links altered FN expression and signaling to epithelial–mesenchymal transition (EMT), angiogenesis, tumor-associated matrix remodeling, and increased tumor invasiveness [7,8,9]. Consistent with these observations, elevated or aberrantly distributed FN has been associated with aggressive disease features and unfavorable clinical outcomes in several malignancies [10].

In addition to its roles in tissue remodeling and cancer biology, FN is increasingly recognized as an important host factor in microbial adhesion and infection. Several bacterial pathogens exploit host-derived FN as a bridging molecule to promote attachment to epithelial or endothelial cells [11]. Bacterial fibronectin-binding proteins interact with FN and thereby facilitate engagement with host–cell receptors such as integrins, which can promote bacterial adhesion and, in some cases, cellular invasion [12,13]. This mechanism has been extensively characterized in *Staphylococcus aureus*, in which fibronectin-binding proteins such as FnBPA and FnBPB contribute to FN-dependent attachment and invasion of non-professional phagocytic cells [12]. Therefore, FN represents not only a structural and signaling component of the ECM but also a host-derived mediator of pathogen–host interactions. FN-binding antibodies may consequently serve as useful research reagents for investigating these FN-dependent biological processes.

Among the various targeting strategies, antibody-based approaches are widely used in biomedical research, diagnostics, and therapeutic development because of their high target selectivity and engineering versatility [14,15]. Rabbits are particularly attractive hosts for antibody discovery because they can generate robust immune responses against conserved or weakly immunogenic antigens and possess a distinctive antibody repertoire diversification mechanism involving somatic hypermutation and gene conversion [16]. These features can contribute to diverse epitope recognition and facilitate the generation of antibodies with favorable binding properties, supporting the widespread use of rabbit antibodies in applications such as immunohistochemistry (IHC)-based biomarker detection, lateral flow assays for infectious diseases, and therapeutic antibody development [15,16,17]. Although polyclonal rabbit antibodies are widely used, their heterogeneous composition, batch-to-batch variability, and dependence on continued animal immunization can limit reproducibility and scalability. Recombinant monoclonal antibody formats can overcome many of these limitations by providing sequence-defined reagents that can be reproducibly produced and further engineered. Among antibody formats, single-chain variable fragments (scFvs), in which the variable heavy (V_H_) and variable light (V_L_) domains are joined by a flexible peptide linker, offer advantages including small molecular size, relatively straightforward bacterial expression, and compatibility with molecular engineering [18]. Importantly, scFvs can be efficiently incorporated into phage display libraries, enabling iterative enrichment and selection of antigen-binding clones from large antibody repertoires [19,20]. Recent studies have further demonstrated the utility of rabbit-derived phage display libraries combined with next-generation sequencing for recombinant monoclonal antibody discovery [21]. In parallel, advances in computational antibody engineering have introduced structure-based, machine-learning, and generative approaches for antibody design and optimization, although experimental display-based selection remains important for the direct identification and validation of antigen-binding molecules [22,23].

In light of the critical role of FN in cancer progression, tissue remodeling, and host–pathogen interactions, together with the distinctive immune repertoire of rabbits, this study aimed to generate and characterize rabbit-derived scFv antibodies against recombinant human FN. By integrating rabbit immunization with phage display, we constructed rabbit-derived scFv libraries, selected a panel of FN-binding scFv clones, and characterized their binding properties using ELISA, Western blotting, and FOPPR-based kinetic analysis. The resulting scFvs provide recombinant FN-binding reagents for future assay development and mechanistic studies, including investigations of FN-mediated host–pathogen interactions.

## 2. Results

### 2.1. Rabbit Immunization and Analysis of the Immune Response

**Figure 1:**
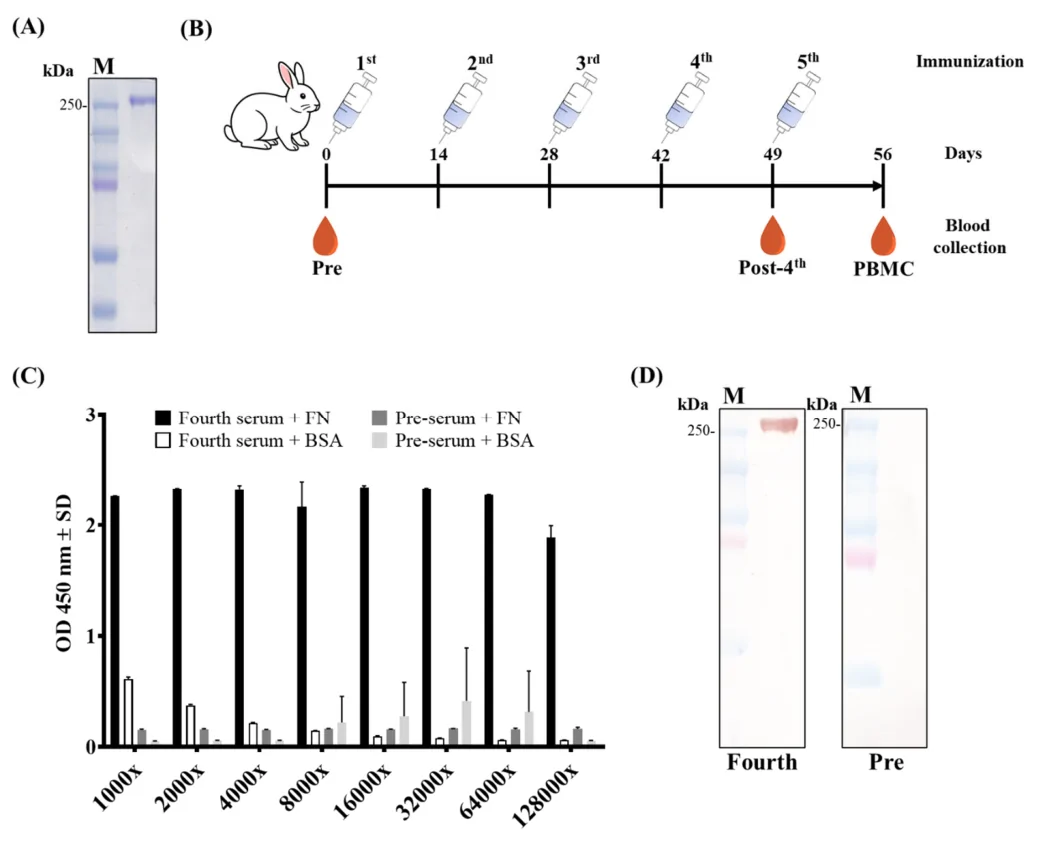
Humoral immune response in a rabbit following fibronectin (FN) protein immunization. (**A**) Recombinant human FN was analyzed by 8% SDS-PAGE and visualized by Coomassie Brilliant Blue staining. (**B**) Schematic timeline of the rabbit immunization protocol. One rabbit was immunized five times with recombinant FN. Blood samples were collected on Day 0 before immunization (Pre) and on Day 49, seven days after the fourth immunization, for serum titration. The fifth immunization was administered on Day 49, and blood was collected on Day 56, seven days after the final immunization, for peripheral blood mononuclear cell (PBMC) isolation. (**C**) ELISA analysis of FN-binding polyclonal antibody responses. Serum samples collected on Days 0 and 49 were serially diluted (1:1000–1:128,000) and tested for binding to immobilized FN or BSA. Data are presented as mean OD_450_ ± SD from duplicate determinations. An OD_450_ value greater than 1.0 was used as the operational threshold. Because the signal remained above this threshold at the highest dilution tested, the endpoint titer was greater than 1:128,000. (**D**) Western blot analysis of FN-binding antibodies in serum samples collected on Days 0 and 49. Recombinant human FN (0.5 μg per lane) was probed with rabbit serum samples, each diluted 1:20,000.

Recombinant FN protein was analyzed by 8% SDS-PAGE and visualized with Coomassie Brilliant Blue staining (Figure 1A). The protein was emulsified with adjuvant and administered to one rabbit. After four immunizations, the humoral immune response was evaluated by ELISA using serial two-fold dilutions of serum collected seven days after the fourth immunization (Figure 1B,C). Post-immunization serum exhibited strong binding to FN, with OD_450_ values remaining above 1.0 even at the highest dilution tested (1:128,000), indicating an endpoint titer greater than 1:128,000. Moderate cross-reactivity with BSA was observed at 1:1000 and 1:2000 dilutions (OD_450_ approximately 0.4–0.8), but the signals decreased to background levels (OD_450_ < 0.2) at higher dilutions. In contrast, pre-immunization serum showed no detectable reactivity toward FN or BSA. The humoral response was further confirmed by Western blot, where serum from the fourth immunization displayed strong FN-binding signals compared with pre-immunization serum (Figure 1D). Collectively, these findings demonstrate that FN immunization successfully elicited a robust antibody response in the rabbit.

### 2.2. Construction of Rabbit scFv Libraries

**Figure 2:**
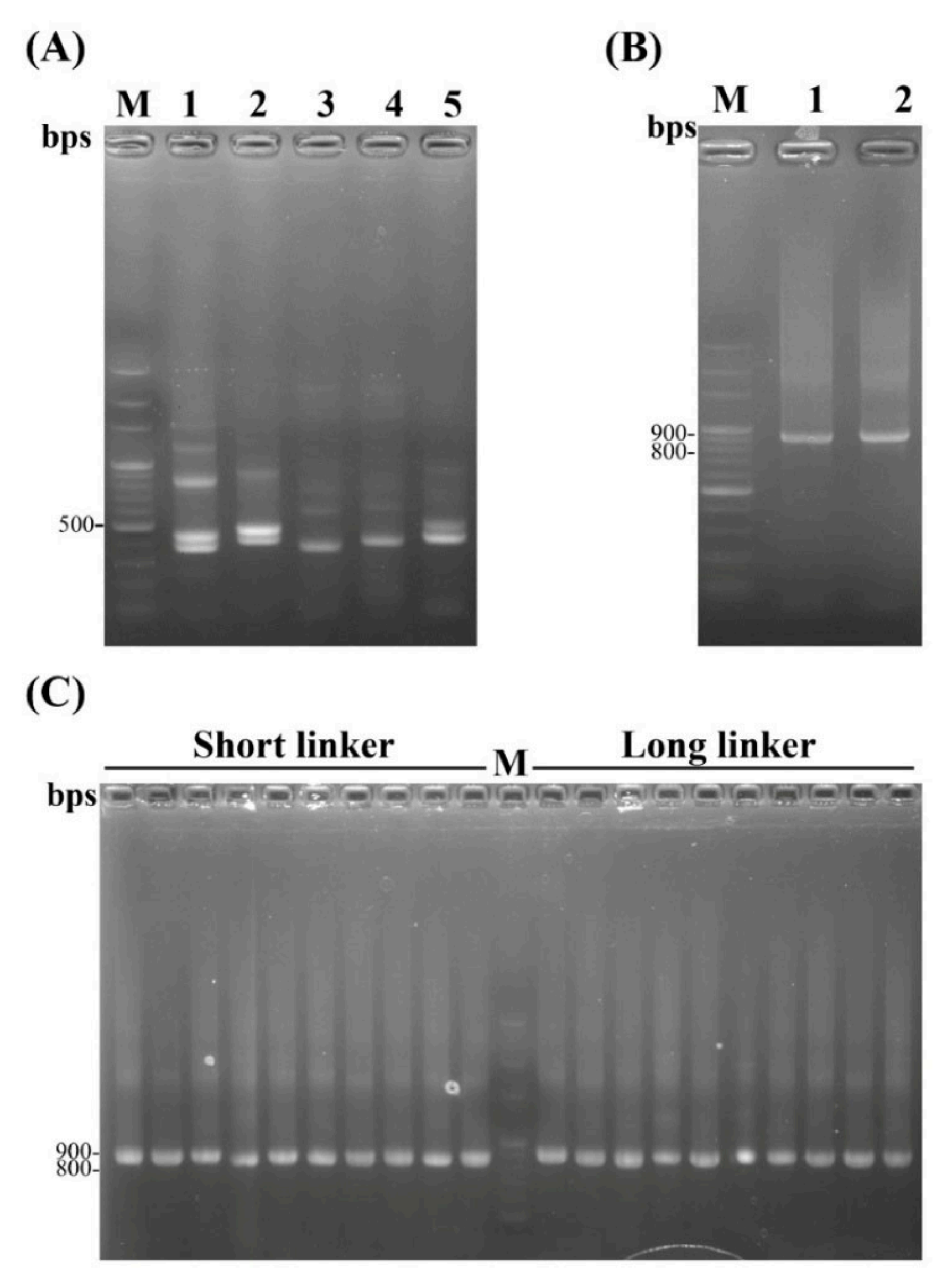
Amplification of rabbit immunoglobulin variable regions and validation of single-chain variable fragment (scFv) library construction. (**A**) PCR amplification of light chain variable regions (V_L_, κ and λ) with short or long linkers and heavy-chain variable regions (V_H_) using rabbit-specific primers. Lane 1: V_L_-κ (short linker); Lane 2: V_L_-κ (long linker); Lane 3: V_L_-λ (short linker); Lane 4: V_L_-λ (long linker); Lane 5: V_H_. (**B**) Overlapping PCR assembly of full-length scFv fragments containing either short or long linkers. (**C**) Colony PCR validation of randomly selected clones. Ten randomly selected colonies from each library were analyzed to confirm the presence of inserts of the expected size.

Seven days after the final immunization, 30 mL of blood was collected, and peripheral blood mononuclear cells (PBMCs) were isolated using Ficoll reagent. Approximately 1.5 × 10^7^ PBMCs were obtained and used for total RNA extraction, which served as the template for cDNA synthesis. The variable regions of the light (V_L_) and heavy (V_H_) chains were amplified by PCR using specific primers (Figure 2A). In a subsequent overlap extension PCR, full-length scFv fragments were assembled by linking V_L_ and V_H_ regions with either a short linker (GGSSRSS) or a long linker (SSGGGGSGGGGGGSSRSS) (Figure 2B). The assembled scFv genes were digested and ligated into the pComb3X vector to construct two phage display libraries. The short-linker library yielded 5.5 × 10^7^ transformants, while the long-linker library yielded 5.0 × 10^7^ transformants. Colony PCR analysis showed that all 10 randomly selected colonies from each library contained inserts of the expected size (Figure 2C).

### 2.3. Selection of FN-Binding scFv Antibodies by Biopanning

**Figure 3:**
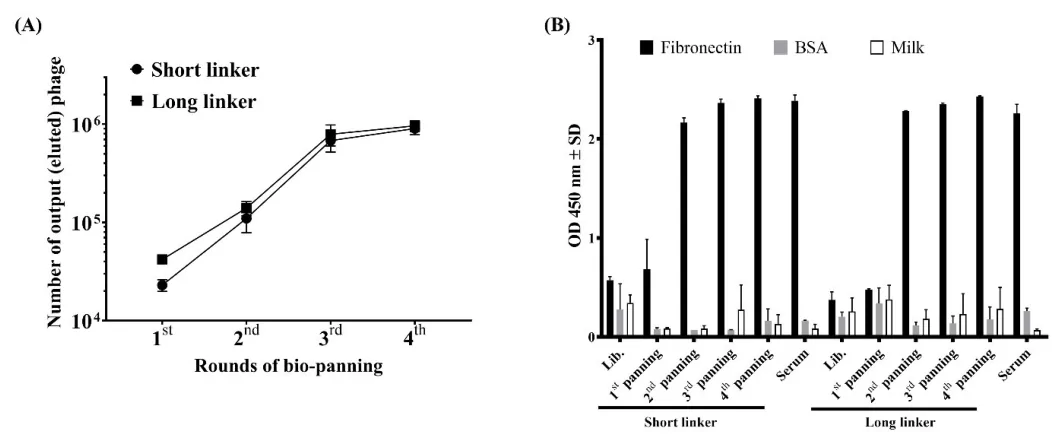
Titers and binding activities of amplified phages during biopanning. (**A**) The two rabbit scFv libraries were subjected to four rounds of biopanning. Eluted phage titers were determined after each round by infecting *E. coli* and calculating colony-forming units (CFU). (**B**) Amplified phages from each round were analyzed for binding to FN by ELISA. Rabbit serum collected on Day 49 was included as a positive control. Data are presented as mean OD_450_ ± SD from duplicate determinations.

Both libraries were subjected to four rounds of biopanning to enrich FN-binding antibodies. After each round, eluted phage titers were determined by colony-forming unit (CFU) counts (Figure 3A). Initial titers were ~10^4^ CFU in both libraries, increasing to ~10^5^ CFU from the second round and nearly 10^6^ CFU by the fourth round. Phage-based ELISA of amplified phages confirmed progressive enrichment of FN-binding clones, with signal intensities increasing markedly after the second round and plateauing thereafter (Figure 3B).

### 2.4. Protein Expression and Binding Assay of Selected scFv Antibodies

**Figure 4:**
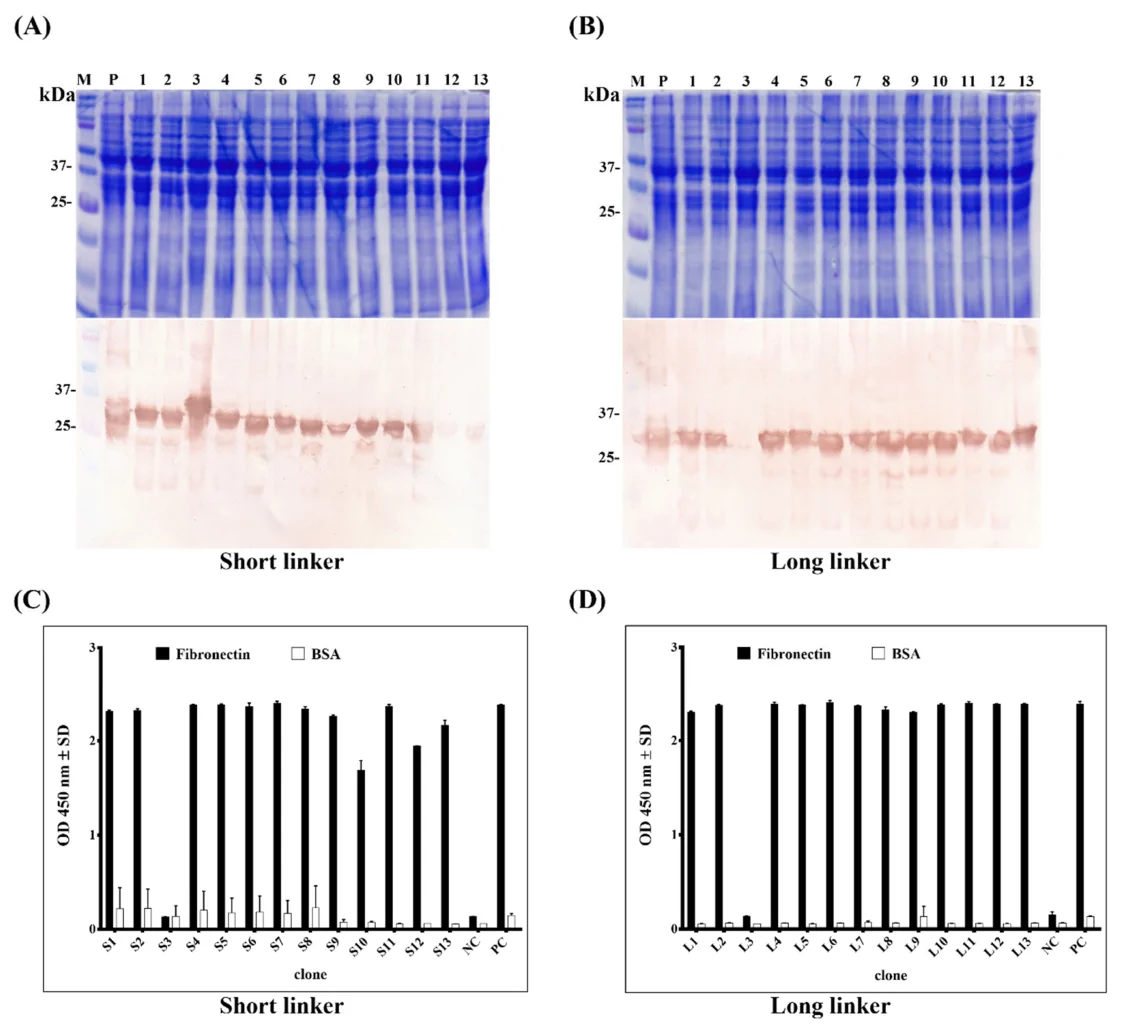
Expression and binding activities of the recombinant scFv antibodies in *E. coli*. After the fourth round of biopanning, 13 clones from each of the short-linker and long-linker libraries were randomly selected for IPTG-induced expression. Whole-cell lysates of the (**A**) short-linker clones FN-S1 to FN-S13 and (**B**) long-linker clones FN-L1 to FN-L13 were analyzed by SDS-PAGE followed by Coomassie blue staining (upper panels), and scFv expression was further confirmed by Western blotting using an anti-HA antibody (lower panels). M, molecular-weight marker; P, a previously confirmed rabbit-derived scFv used as a positive control for expression. The binding activities of scFv antibodies expressed in bacterial lysates from the (**C**) short-linker and (**D**) long-linker libraries were evaluated by ELISA against fibronectin. An unrelated rabbit-derived scFv and post-immunization serum collected on Day 49 were used as the negative and positive controls, respectively. NC, negative control; PC, positive control. Data are presented as mean OD_450_ ± SD from duplicate wells.

Thirteen clones were randomly selected from each library after the fourth round of biopanning and expressed in *E. coli* TOP10F’ cells. Following IPTG induction, cell lysates were analyzed by SDS-PAGE and Western blot (Figure 4A,B). Although distinct bands were not apparent on SDS-PAGE, Western blot analysis confirmed expression of scFv antibodies with the expected molecular weight (25–37 kDa) in all clones except clone 3 from the long-linker library, which showed no detectable expression. Binding activity was subsequently evaluated by ELISA (Figure 4C,D). Among the evaluable clones, all showed detectable binding to FN except FN-S3. The binding activity of FN-L3 could not be reliably evaluated because its premature stop codon prevented production of the complete V_H_ domain and the C-terminal HA tag required for detection.

### 2.5. Sequence Analysis and Classification of Selected scFv Antibodies

Sequencing analysis grouped the clones based on identical sequences, yielding FN-S1 (5/13; 38.5%), FN-S3 (1/13; 7.7%), FN-S4 (3/13; 23.1%), FN-S6 (2/13; 15.4%), and FN-S8 (2/13; 15.4%) from the short-linker library; and FN-L1 (7/13; 53.8%), FN-L3 (1/13; 7.7%), FN-L5 (4/13; 30.8%), and FN-L13 (1/13; 7.7%) from the long-linker library (Table 1). Separate comparison of the V_L_ and V_H_ sequences showed that no identical V_H_ sequence was paired with different V_L_ sequences or shared between the short- and long-linker libraries. Likewise, no identical V_L_ sequence was paired with different V_H_ sequences. Notably, FN-L3 contained a premature TGA stop codon at the second codon of V_H_ FR1, preventing completion of the V_H_ domain and translation of the C-terminal HA and His tags. Consequently, no full-length tagged FN-L3 product was detected by Western blot (Figure 4B), and its FN-binding activity could not be evaluated by ELISA (Figure 4D). Deduced amino acid sequences of the V_L_ and V_H_ regions were compared with their assigned germline sequences to assess amino acid divergence (Table 2). Complementarity-determining regions (CDRs) generally showed greater divergence than framework regions (FRs), particularly in the V_H_ domain. In the V_H_ domain, divergence ranged from 25–50% in CDR1, 57–75% in CDR2, and 0–27% in CDR3, with total CDR divergence ranging from 22–37%. In the V_L_ domain, divergence ranged from 0–50% in CDR1, 0–33% in CDR2, and 0–10% in CDR3, with total CDR divergence ranging from 0–25%. Divergence in the FRs was generally lower: V_H_ FR1 (0–16%), FR2 (6–35%), FR3 (8–32%), and FR4 (0%); V_L_ FR1 (4–32%), FR2 (0–18%), FR3 (0–24%), and FR4 (0%). Overall, total FR divergence ranged from 4–23% in V_H_ and 2–21% in V_L_.

### 2.6. Binding Activity Assay of Selected Rabbit scFv Antibodies

**Figure 5:**
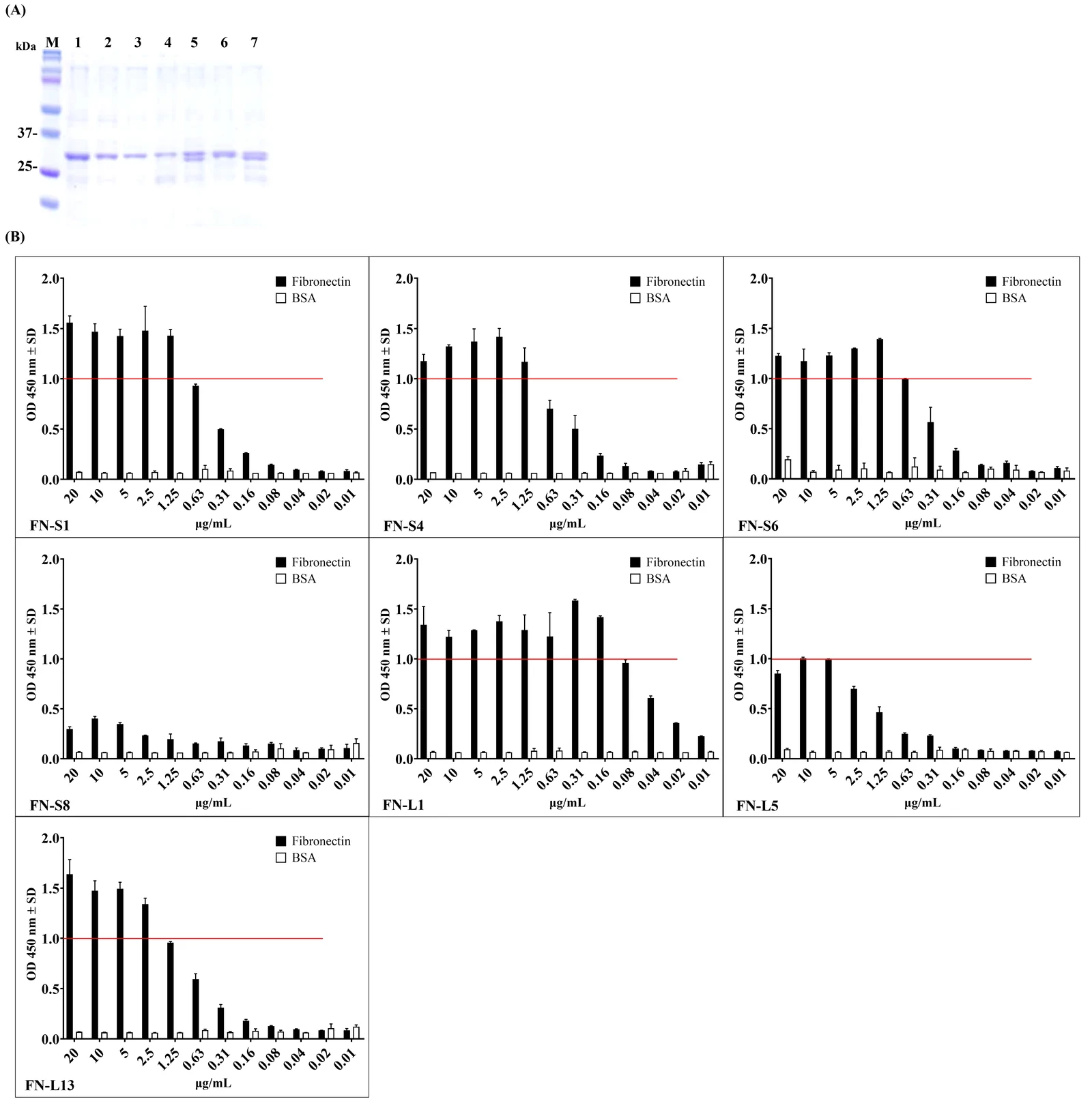
Purification and ELISA-based binding activity of selected scFv antibodies. (**A**) Selected scFv antibodies were purified from *E. coli* using Ni^2+^ Sepharose and analyzed by SDS-PAGE with Coomassie blue staining. Lane 1: FN-S1; Lane 2: FN-S4; Lane 3: FN-S6; Lane 4: FN-S8; Lane 5: FN-L1; Lane 6: FN-L5; Lane 7: FN-L13. (**B**) Binding activities of purified scFv antibodies were determined by ELISA using two-fold serial dilutions (20 μg/mL to 0.01 μg/mL). The horizontal red line indicates an OD_450_ value of 1.0. Data are presented as mean OD_450_ ± SD from duplicate determinations.

Selected scFv antibodies were expressed and purified using Ni^2+^ Sepharose chromatography, excluding FN-S3 (non-binding on ELISA) and FN-L3 (premature stop codon). Seven purified scFv antibodies were analyzed by SDS-PAGE, showing bands around 27 kDa with minor size variations (Figure 5A). Western blotting with an anti-HA antibody showed that the major bands, as well as additional HA-reactive species observed in several scFv preparations, retained the C-terminal HA tag. The double-band patterns were most evident for FN-L1 and FN-L13, supporting that these additional species were derived from the recombinant scFv constructs rather than unrelated co-purified proteins (Supplementary Figure S1). The purification yields of the seven FN-binding constructs ranged from 0.25 to 0.81 mg per liter of bacterial culture (Table 3). ELISA titration assays revealed differential binding activities (Figure 5B). FN-L1 displayed the strongest activity, maintaining OD_450_ > 1.0 at concentrations as low as 0.16 μg/mL, with gradual signal decline upon dilution. Other clones showed detectable signals (OD_450_ > 1.0) at varying concentrations: FN-S1 (2.5 μg/mL), FN-S4 (1.25 μg/mL), FN-S6 (1.25 μg/mL), FN-L5 (10 μg/mL), and FN-L13 (2.5 μg/mL). In contrast, FN-S8 showed weak ELISA reactivity across the tested concentration range.

Fiber-optic particle plasmon resonance (FOPPR)-based kinetic analysis revealed that the seven purified fibronectin-binding scFvs exhibited apparent equilibrium dissociation constant (*K_D_*_,app_) values ranging from 11.21 to 432.06 nM (Table 4; Supplementary Files). FN-L13 and FN-S4 showed the lowest *K_D_*_,app_ values of 11.21 and 15.45 nM, respectively, and also exhibited the slowest dissociation rates among the tested constructs. FN-S1 and FN-S6 showed intermediate *K_D_*_,app_ values of approximately 100 nM, whereas FN-L5, FN-L1, and FN-S8 exhibited values of 196.34, 358.71, and 432.06 nM, respectively. In addition, preliminary FOPPR-based sequential-binding analysis was performed using FN-L13 as the reference scFv. FN-L13 was selected because it provided a relatively high purification yield and exhibited the lowest *K_D,app_* among the long-linker constructs. Following the binding of FN-L13 to immobilized fibronectin, subsequent injection of each of the other six scFvs produced no further detectable decrease in the sensor response (Supplementary Figure S2 and Table S1). These results suggest apparent competition between each of the six scFvs and FN-L13 and indicate that the seven clones may recognize the same or closely overlapping epitope region on fibronectin.

To further assess the oligomeric states of the purified scFvs, analytical SEC was performed on all seven constructs. BSA (~66 kDa), used as a molecular-size reference, eluted at approximately 13.55 min, whereas the major scFv-associated peaks eluted substantially later, at approximately 20.17–20.82 min. These retention profiles were consistent with the scFvs being predominantly monomeric species, with expected molecular masses of approximately 26–32 kDa, rather than predominantly dimeric species (~52–64 kDa), under the conditions tested. Minor earlier-eluting peaks were observed in several preparations, suggesting the presence of small amounts of higher-molecular-weight species (Supplementary Figure S3).

### 2.7. Western Blot Analysis of Rabbit scFv Reactivity Toward FN and Mouse Plasma

**Figure 6:**
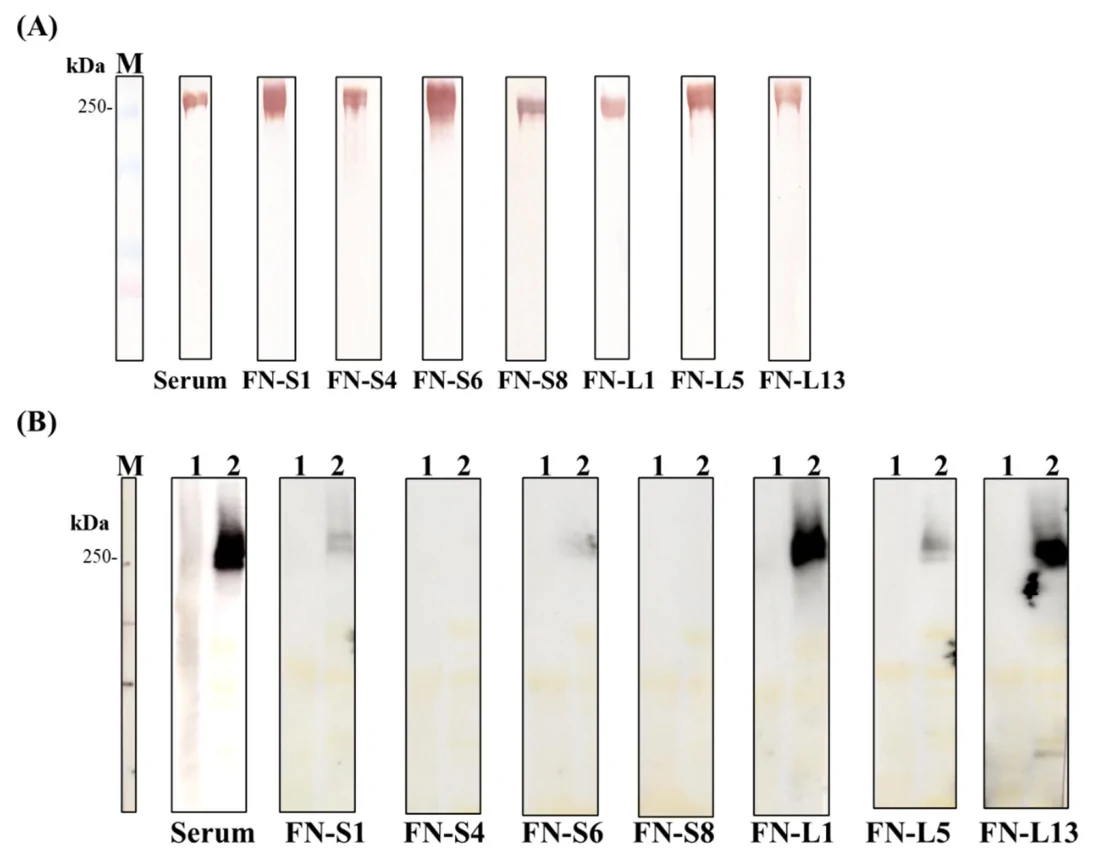
Western blot analysis of scFv antibodies against FN protein and mouse plasma. (**A**) Recombinant FN protein (0.5 μg per lane) was separated by SDS-PAGE, transferred to PVDF membranes, and probed with bacterial lysates containing the selected scFv antibodies. (**B**) Twenty micrograms of total protein from mouse muscle lysate (Lane 1) and plasma (Lane 2) were separated by SDS-PAGE, transferred to PVDF membranes and probed with seven purified scFv antibodies. Rabbit serum collected on Day 49 was used as a positive control. The cropped blot panels were aligned to a common molecular-weight scale, with a single molecular-weight marker shown on the left of each panel. Lane M, molecular-weight marker.

Binding reactivity of the selected scFv antibodies was further examined by Western blot using scFv-containing bacterial lysates (Figure 6A). The seven purified constructs were subsequently evaluated for cross-species reactivity using mouse plasma, with mouse muscle lysate included as a tissue lysate control (Figure 6B). FN-S4 and FN-S8 displayed no reactivity, whereas the other clones exhibited distinct signal intensities. FN-S1, FN-S6, and FN-L5 produced weaker signals, while FN-L1 and FN-L13 produced stronger signals, indicating relatively stronger reactivity under the conditions tested. None of the clones showed detectable reactivity with mouse muscle lysate under the conditions tested.

### 2.8. Binding Selectivity and Cross-Reactivity Analysis of Rabbit scFv Antibodies Against Selected ECM-Associated and Plasma Proteins

**Figure 7:**
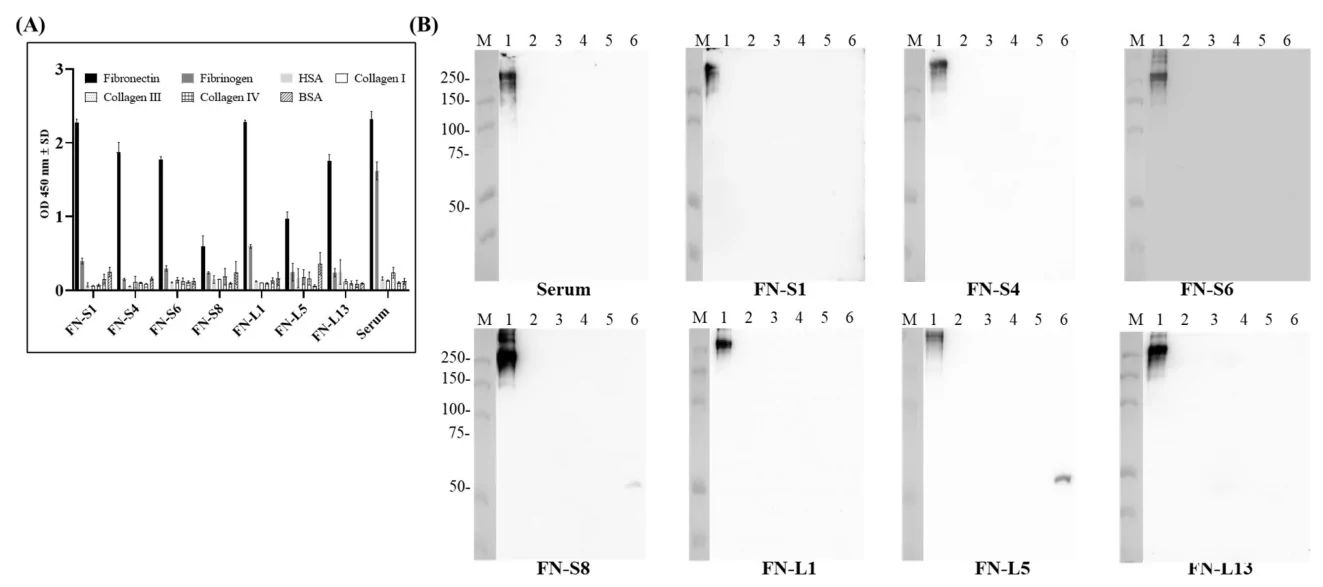
Binding selectivity and cross-reactivity of rabbit scFv antibodies toward FN and selected extracellular matrix (ECM)-associated and plasma proteins. (**A**) The binding profiles of seven purified scFv antibodies (FN-S1, FN-S4, FN-S6, FN-S8, FN-L1, FN-L5, and FN-L13) and post-immunization rabbit serum were evaluated by ELISA against FN, fibrinogen, human serum albumin (HSA), collagens I, III, and IV, and BSA. BSA was included as an additional negative-control protein. Binding was detected by measuring absorbance at 450 nm. Data are presented as mean OD_450_ ± SD from duplicate determinations. (**B**) The purified scFv antibodies and post-immunization rabbit serum were further evaluated by Western blotting against FN (lane 1), fibrinogen (lane 2), HSA (lane 3), and collagens I (lane 4), III (lane 5), and IV (lane 6) under denaturing conditions. Lane M, molecular-weight marker.

The binding selectivity of the purified scFvs was further evaluated against selected ECM-associated and plasma proteins, including fibrinogen, human serum albumin (HSA), and collagens I, III, and IV, using ELISA and Western blotting. BSA was included as an additional negative-control protein in the ELISA. In the ELISA, all seven scFvs showed preferential binding to FN, whereas reactivity toward HSA, BSA, and the tested collagens remained generally low (Figure 7A). FN-S1 and FN-L1 showed detectable reactivity toward fibrinogen (OD_450_ values of approximately 0.4 and 0.6, respectively), while the remaining scFvs exhibited little or no detectable fibrinogen binding. The post-immunization rabbit serum also showed strong reactivity toward FN and detectable reactivity toward fibrinogen. SDS-PAGE analysis of the protein preparations showed heterogeneous banding patterns for the collagen samples, including lower-molecular-weight species in the collagen IV preparation (Supplementary Figure S4). Western blot analysis showed predominant reactivity toward FN for most of the purified scFvs under denaturing conditions (Figure 7B). FN-S8 showed weak reactivity with an approximately 50–60 kDa species present in the collagen IV preparation, whereas FN-L5 showed a more prominent signal at a similar molecular weight. Little or no detectable off-target reactivity was observed for the remaining scFvs under the conditions tested. The post-immunization rabbit serum showed predominant reactivity toward FN under the same conditions.

## 3. Discussion

FN plays a pivotal role in ECM integrity and in regulating cellular processes such as adhesion, migration, remodeling, angiogenesis, and tumor-associated matrix organization [2,3]. Aberrant FN expression and signaling have also been associated with tumor progression and inflammatory microenvironments [7,8,9,10]. In this study, immunization with recombinant human FN elicited a strong humoral response in a rabbit despite the high sequence similarity between human FN (UniProt: P02751) and the predicted rabbit FN sequence (NCBI RefSeq: XP_008252979.1) (Figure 1). This finding is consistent with the utility of rabbits for antibody discovery against conserved antigens and with the extensive diversification of the rabbit antibody repertoire [16]. These features make rabbits a useful host for generating antibodies against highly conserved proteins. Nevertheless, because the antibody libraries in this study were generated from a single immunized rabbit, the present findings should be interpreted as proof-of-concept evidence and may not capture inter-animal variability in immune repertoire composition or antibody recovery.

To harness this strong immune response for recombinant antibody development, we constructed two rabbit-derived scFv libraries. Rabbit monoclonal antibody generation has historically been limited by the technical challenges associated with rabbit hybridoma systems, whereas phage display provides a flexible in vitro platform for recovering antigen-binding antibodies from immune or synthetic repertoires [16,19,20]. Recent studies using rabbit antibody libraries have further demonstrated the feasibility of combining rabbit immune repertoires with phage display and next-generation sequencing for recombinant antibody discovery [21]. In our study, one hyperimmunized rabbit was used to construct two scFv phage display libraries containing 5.5 × 10^7^ and 5.0 × 10^7^ transformants, respectively (Figure 2). Library quality was initially evaluated by colony PCR using 10 randomly selected colonies from each library. Because unselected clones were not sequenced, the proportion of complete in-frame constructs across the entire libraries could not be determined. Through two to four rounds of biopanning, FN-binding clones were progressively enriched and several clones with detectable binding activity were recovered within approximately one month, consistent with our previous experience using an immune phage display strategy in chickens (Figure 3 and Figure 4) [24]. The immune-library approach therefore enabled relatively rapid recovery of antigen-reactive clones without requiring the prolonged workflows typically associated with conventional hybridoma generation [25,26]. Nevertheless, independent amplification and combinatorial assembly of the V_H_ and V_L_ repertoires do not preserve the native V_H_-V_L_ pairings generated during in vivo affinity maturation [27,28]. This loss of native pairing, together with the relatively modest library sizes, may have restricted the sequence diversity and binding properties of the recovered clones [29]. Future strategies that preserve native V_H_-V_L_ pairing or incorporate affinity-maturation approaches, such as chain shuffling, may facilitate the recovery of antibodies with improved binding and biophysical properties [30].

The variable regions of V_L_ and V_H_ generate antibody diversity through V(D)J recombination, junctional variation, somatic hypermutation (SHM), and heavy/light chain pairing in most vertebrates [31]. In rabbits, however, the heavy-chain repertoire is generated from a relatively restricted set of germline *V_H_* genes, with *V_H_1* being predominantly utilized, followed by extensive diversification through junctional modification, somatic hypermutation, and gene conversion [16,32,33]. Recent repertoire profiling has further demonstrated the considerable sequence diversity accessible in rabbit antibody libraries [21]. In our analysis, substantial amino acid divergence from the assigned germline sequences was observed not only within the CDRs but also in several framework regions, particularly V_H_ FR2 and V_H_ FR3 (Table 2). Although the CDRs are the principal determinants of antigen recognition, framework residues can influence V_H_-V_L_ interdomain orientation, antibody flexibility, affinity, and stability [34]. The functional consequences of the framework-region divergence observed in these clones were not directly evaluated and therefore remain to be determined.

Complementary to experimental affinity-maturation strategies, recent advances in computational antibody engineering have enabled structure-guided sequence optimization, machine-learning-assisted affinity prediction, and generative design of antibody and nanobody candidates [22,23]. Such approaches can facilitate exploration of sequence space and help prioritize mutations for experimental testing. However, computational predictions still require empirical validation because antibody binding and developability can be influenced by antigen conformation, epitope accessibility, interdomain orientation, conformational dynamics, and other biophysical properties that remain difficult to predict comprehensively. Thus, computational antibody design and display-based experimental selection are best viewed as complementary approaches. For the FN-binding scFvs identified here, future sequence- and structure-guided modeling could be used to prioritize mutations for affinity maturation or improvement of biophysical properties, followed by experimental validation.

Although the libraries showed progressive enrichment during biopanning, FN-S3 showed no detectable binding activity, whereas the binding activity of FN-L3 could not be reliably evaluated because of its premature stop codon (Figure 4). Such nonproductive or non-evaluable clones may result from nonspecific carryover during selection, differences in propagation efficiency, avidity associated with phage-displayed antibody fragments, or conformational differences between phage-displayed and soluble scFvs expressed in *E. coli*. In particular, FN-L3 contained a premature TGA stop codon at the second codon of V_H_ FR1. Given the V_L_-linker-V_H_ construct orientation, the V_L_-linker region may still be translated, whereas the downstream V_H_ region and C-terminal tags would not be produced. Therefore, the selection of FN-L3 following biopanning does not establish that it encodes a functional soluble scFv. Reconstruction and subsequent characterization of a full-length FN-L3 construct will be required to determine whether it can form a functional FN-binding molecule. The remaining seven clones were purified and further evaluated by ELISA titration assays (Figure 5). Western blotting with an anti-HA antibody further showed that the major bands and additional HA-reactive species observed in several scFv preparations retained the C-terminal HA tag, with the double-band patterns being particularly evident for FN-L1 and FN-L13 (Supplementary Figure S1). This finding suggests that the additional bands were scFv-derived species rather than unrelated co-purified proteins. The banding patterns may reflect heterogeneous processing or limited proteolysis during bacterial expression, although their precise molecular identities remain undetermined. Together with the SEC results showing predominantly monomeric species, these findings suggest that the double-band patterns are unlikely to reflect predominant stable dimer formation. Notably, the 7-aa linker used in the short-linker constructs falls within the linker-length range previously associated with intermolecular V_H_-V_L_ association and the formation of diabody-like or higher-order assemblies [18,35]. To examine whether such oligomerization occurred in the present constructs, the seven purified scFvs were further analyzed by analytical size-exclusion chromatography (SEC). The major scFv-associated populations eluted substantially later than the 66-kDa BSA reference and showed retention behavior consistent with predominantly monomeric scFv species, whereas only minor earlier-eluting higher-molecular-weight populations were observed (Supplementary Figure S3). These findings indicate that stable dimeric or higher-order assemblies were not the predominant forms under the SEC conditions tested and are therefore unlikely to account for the majority of the observed binding responses through avidity. Nevertheless, because SEC retention behavior alone does not provide an absolute molecular-mass determination, and minor higher-molecular-weight populations were detected, limited oligomerization or concentration-dependent self-association under other assay conditions cannot be completely excluded. The non-monotonic ELISA responses observed at higher scFv concentrations may instead reflect assay-dependent effects such as steric limitations, aggregation, or changes in the fraction of functionally active protein. Because endpoint ELISA is sensitive to antigen presentation and concentration-dependent assay effects, these titration profiles should not be interpreted as direct measures of affinity [36].

FOPPR enables real-time monitoring of binding responses and has been applied to kinetic characterization of antigen–antibody interactions [37,38]. The resulting apparent equilibrium dissociation constants *K_D,app_* ranged from 11.21 to 432.06 nM (Table 4), indicating that the selected panel comprised FN-binding constructs with nanomolar-to-submicromolar apparent affinities. FN-L13 and FN-S4 exhibited the lowest *K_D,app_* values of 11.21 and 15.45 nM, respectively, which were associated with their relatively slow dissociation rates. In particular, FN-L13 showed the lowest *K_D,app_* among the long-linker constructs. The affinity ranking determined by FOPPR did not fully parallel the signal intensities observed in the ELISA titration assay. For example, FN-L1 showed relatively strong apparent binding in ELISA but exhibited a *K_D,app_* of 358.71 nM, whereas FN-L13 displayed the lowest FOPPR-derived *K_D,app_*. This discrepancy may reflect differences in surface chemistry, antigen density and orientation, the fraction of functionally active scFv, protein stability, steric effects, aggregation, and possible avidity. Although both ELISA and FOPPR are surface-based assays, endpoint ELISA does not resolve association and dissociation kinetics, whereas FOPPR enables kinetic parameters to be derived from real-time binding responses. Therefore, ELISA signal intensity alone cannot reliably predict kinetic affinity. Analytical SEC indicated that the purified scFvs were predominantly monomeric, reducing, but not completely eliminating, the possibility that oligomerization or avidity influenced the FOPPR responses (Supplementary Figure S3). The kinetic values are therefore conservatively reported as apparent affinities (*K_D,app_*), because surface immobilization, ligand density and orientation, surface rebinding, and residual sample heterogeneity may still influence the measured interaction parameters. Orthogonal kinetic analysis using SPR or BLI could provide further validation of these findings.

Preliminary FOPPR-based sequential-binding analysis indicated that all six tested scFvs showed apparent competition with FN-L13, suggesting that the seven FN-binding clones may recognize the same or closely overlapping epitope region on fibronectin (Supplementary Figure S2 and Table S1). However, the absence of a further decrease in sensor response does not demonstrate recognition of identical amino acid residues, because partially overlapping epitopes, steric interference, or surface-dependent effects may produce similar results. Reciprocal pairwise competition and complementary epitope-mapping approaches will therefore be required to define their epitope relationships more precisely. The apparent convergence of these clones may limit their direct use as matched capture and detection reagents in sandwich immunoassays, which generally require simultaneous binding to nonoverlapping or sufficiently separated epitopes [39]. An additional practical limitation is that all seven scFvs carry the same C-terminal His and HA tags, which would complicate tag-based discrimination between capture and detection reagents in the current format. Future assay development may therefore require differential labeling or reformatting of selected clones, such as using an untagged or Fc-fused capture reagent paired with a biotinylated or alternatively tagged detection scFv. Alternatively, the present scFvs could be paired with independently generated anti-FN antibodies recognizing nonoverlapping epitopes. Such approaches should be evaluated experimentally before their suitability for sandwich-based detection can be established.

Because human-derived plasma samples were not evaluated in the present study, mouse plasma was used to examine cross-species reactivity under Western blot conditions, given the high sequence conservation between human and mouse fibronectin (~91% amino acid identity) [40]. Five of the seven purified constructs showed detectable reactivity with a high-molecular-weight band at the expected position of FN in mouse plasma, although the signal intensities and banding patterns varied among clones. FN-S4 and FN-S8 showed no reactivity, whereas the remaining constructs displayed distinct binding profiles, which may reflect differences in epitope accessibility or binding properties. These findings demonstrate that several of the rabbit-derived scFvs can recognize a high-molecular-weight species in mouse plasma consistent with endogenous FN under denaturing Western blot conditions, but they should not be interpreted as evidence of binding to native human plasma FN. Because FN is broadly distributed in normal tissues and biological fluids and exists in multiple context-dependent isoforms, including EDA- and EDB-containing variants, total FN detection alone may have limited disease specificity. Further validation using human-derived samples and orthogonal binding assays will therefore be required before the utility of these antibodies in analytical or diagnostic applications can be assessed.

Additional cross-reactivity analysis against selected ECM-associated and plasma proteins further showed that the binding selectivity of the selected scFvs was clone-dependent (Figure 7). In ELISA, all seven scFvs showed preferential binding to FN, with generally low reactivity toward HSA and collagens I, III, and IV. FN-S1 and FN-L1 showed detectable reactivity toward fibrinogen. In contrast, under Western blot conditions, FN-S8 and FN-L5 showed reactivity with an approximately 50–60 kDa species present in the collagen IV preparation, with the signal being more prominent for FN-L5. The molecular identity of this reactive species remains unclear and requires further investigation. The differences between the ELISA and Western blot results suggest that off-target reactivity may depend, at least in part, on antigen presentation and assay format. These findings emphasize that the molecular selectivity of individual scFvs should be evaluated separately rather than assuming uniform specificity across the entire panel. Because the tested proteins represent only a limited subset of ECM-associated and plasma proteins, broader cross-reactivity profiling would be required before future analytical applications.

Beyond their potential use as FN-detection reagents, FN-binding scFvs may serve as useful tools for studying FN-associated biological mechanisms. FN is increasingly recognized as a host-derived bridging molecule in bacterial adhesion, in which bacterial fibronectin-binding proteins interact with FN and connect bacterial surfaces to host–cell receptors such as integrins [11,12]. This mechanism has been particularly well described in *Staphylococcus aureus*, where fibronectin-binding proteins mediate adhesion to and invasion of non-professional phagocytic cells through FN–integrin interactions [13]. Although the present study did not directly evaluate bacterial adhesion or invasion, the FN-binding scFvs generated here may provide useful reagents for probing FN-mediated host–pathogen interactions. In future studies, adhesion or invasion assays using relevant bacterial models may help determine whether these scFvs can interfere with FN-dependent bacterial attachment. Such experiments would further clarify whether these antibodies are limited to FN detection or may also have value as functional tools for investigating FN-dependent bacterial adhesion mechanisms.

In summary, this study generated and characterized a panel of rabbit-derived scFv antibodies against recombinant human FN. FOPPR-based kinetic analysis yielded *K_D,app_* values ranging from 11.2 to 432 nM, with FN-L13 displaying the lowest value of 11.2 nM. Analytical SEC indicated that the purified scFvs were predominantly monomeric, with minor higher-molecular-weight species. Five constructs showed reactivity with a high-molecular-weight band in mouse plasma consistent with FN under Western blot conditions, while cross-reactivity analysis showed overall preferential binding to FN, with clone-dependent and assay-dependent off-target reactivity. Further validation using human-derived samples, broader specificity profiling, and orthogonal binding approaches will be required before their analytical utility can be established. Nevertheless, these scFvs provide recombinant FN-binding reagents for future assay development and mechanistic studies, including investigations of FN-associated host–pathogen interactions.

## 4. Materials and Methods

### 4.1. Ethical Approval of Animal Experiments

All animal procedures were conducted in accordance with institutional guidelines and approved by the Institutional Animal Care and Use Committee (IACUC) of National Yang Ming Chiao Tung University (approval code: 111018A; approved on 18 July 2022 and valid until 17 July 2024). One male New Zealand White rabbit (*Oryctolagus cuniculus*), six months of age, was used for immunization. The rabbit was housed under standard laboratory conditions at the Laboratory Animal Center of National Yang Ming Chiao Tung University, Chiaotung Campus.

### 4.2. Rabbit Immunization

Recombinant human FN protein was purchased from Healthgen Biotechnology (Wuhan Healthgen Biotechnology Corp., Wuhan, China). For the primary immunization, 500 µg of FN in 0.25 mL phosphate-buffered saline (PBS) was emulsified with an equal volume of complete Freund’s adjuvant (Sigma-Aldrich, St. Louis, MO, USA) and administered via subcutaneous injection. Subsequent booster immunizations (2nd to 4th) consisted of 100 µg of FN in 0.25 mL PBS emulsified with incomplete Freund’s adjuvant (Sigma-Aldrich, St. Louis, MO, USA). The second, third, and fourth immunizations were administered at 14-day intervals on Days 14, 28, and 42, respectively. After serum collection on Day 49, the fifth and final immunization was administered using the same antigen and adjuvant formulation.

### 4.3. Construction of Rabbit Phage Display scFv Libraries

Rabbit scFv antibody libraries were constructed as previously described [41]. Briefly, 30 mL of blood was collected 7 days after the final immunization, and peripheral blood mononuclear cells (PBMCs) were isolated using Ficoll-Paque™ PLUS density gradient medium (Cytiva Sweden AB, Uppsala, Sweden). Total RNA was extracted from PBMCs with TRIzol reagent (Invitrogen, Carlsbad, CA, USA) and 10 µg of RNA was reverse-transcribed into cDNA. The cDNA served as the template for amplification of variable light (V_L_; κ and λ) and heavy (V_H_) chain genes using rabbit immunoglobulin-specific primers [41]. The amplified V_L_ and V_H_ fragments were assembled into full-length scFv genes using either a 7-amino acid linker (GGSSRSS) or an 18-amino acid linker (SSGGGGSGGGGGGSSRSS). The scFv constructs were digested with *Sfi*I (New England Biolabs, Ipswich, MA, USA) and ligated into the pComb3X vector. Recombinant vectors were electroporated into *Escherichia coli* (*E. coli*) ER2738 using a MicroPulser (Bio-Rad, Hercules, CA, USA). A subset of transformants was plated on LB agar containing 50 µg/mL ampicillin to estimate library size, while the remainder was cultured in super broth and infected with 10^12^–10^13^ plaque-forming units (PFU) of VCS-M13 helper phage. Following overnight incubation at 37 °C, recombinant phages were harvested by centrifugation and precipitated with 4% polyethylene glycol (PEG) 8000 and 3% NaCl on ice for at least 30 min. Pellets were resuspended in PBS containing 1% bovine serum albumin (BSA) and 20% glycerol for storage and subsequent use.

### 4.4. Selection of FN-Binding scFv Antibodies by Biopanning

Biopanning was carried out using 96-well microplates (Corning 9018; Corning Inc., Corning, NY, USA). Wells were coated with 0.5 µg of recombinant FN protein and incubated overnight at 4 °C. After blocking with 3% BSA in PBS for 1 h at 37 °C, recombinant phages (10^11^–10^12^ PFU) were added and incubated at 37 °C for 2 h. Unbound phages were removed with PBST (PBS containing 0.05% Tween 20). Bound phages were eluted with 0.1 M glycine-HCl (pH 2.2) and neutralized with 2 M Tris base. The eluted phages were used to infect *E. coli* ER2738 cells. A portion of infected cells was plated on LB agar containing 50 µg/mL ampicillin to determine phage titers, while the remainder was amplified overnight. Recombinant phages were then harvested as described above and used for subsequent rounds of biopanning.

### 4.5. Protein Expression and Purification of Rabbit-Derived scFv Antibody

Recombinant plasmids obtained after the final round of biopanning were purified and subsequently transformed into *E. coli* TOP10F’ cells for protein expression. A single colony was inoculated into super broth containing 20 mM MgCl_2_ and 50 µg/mL ampicillin and cultured overnight. The culture was diluted 1:100 in fresh super broth and incubated at 37 °C for 8 h, followed by induction with 1 mM IPTG and overnight incubation. Bacterial cells were harvested by centrifugation and resuspended in His-binding buffer (20 mM sodium phosphate, 0.5 M NaCl, 20 mM imidazole, pH 7.4). The bacterial suspension was lysed by sonication to release recombinant scFv proteins, which were subsequently purified using Ni^2+^ Sepharose (Cytiva, Global Life Sciences Solutions USA LLC, Wilmington, DE, USA) according to the manufacturer’s instructions. Purified scFv antibody concentrations were determined using a BCA assay (Thermo Fisher Scientific Inc., Waltham, MA, USA).

### 4.6. Western Blot Analysis

Recombinant human FN protein (0.5 μg per lane) was analyzed to evaluate FN binding, whereas mouse muscle lysate (20 µg total protein per lane; iReal Biotechnology Inc., Hsinchu, Taiwan) and mouse plasma (20 µg total protein per lane; iReal Biotechnology Inc., Hsinchu, Taiwan) were analyzed to assess reactivity with tissue and plasma samples. In separate experiments, FN (0.5 μg per lane), fibrinogen (0.5 μg per lane; NJDULY, Nanjing, China), human serum albumin (HSA; 0.5 μg per lane; Sigma-Aldrich, St. Louis, MO, USA), and collagens I, III, and IV (0.5 μg per lane; SouthernBiotech, Birmingham, AL, USA) were analyzed for cross-reactivity. All samples were separated by SDS-PAGE and transferred onto polyvinylidene fluoride (PVDF) membranes. Membranes were blocked with 5% skim milk in PBS for 1 h at 25 °C. For analysis of the rabbit immune response, membranes containing recombinant FN were incubated with rabbit serum collected on Day 49, seven days after the fourth immunization (1:20,000 dilution) for 1 h at 25 °C. After three washings using PBST, bound antibodies were detected using horseradish peroxidase (HRP)-conjugated goat anti-rabbit IgG at a 1:10,000 dilution (iReal Biotechnology Inc., Hsinchu, Taiwan).

To detect scFv antibodies expressed in bacterial lysates, membranes were probed with rabbit anti-HA antibody (iReal Biotechnology Inc., Hsinchu, Taiwan) at a 1:5000 dilution, followed by HRP-conjugated goat anti-rabbit IgG (iReal Biotechnology Inc., Hsinchu, Taiwan). Purified scFv preparations were also analyzed by Western blotting using rabbit anti-HA antibody to evaluate HA-reactive protein species. For analysis of FN binding, bacterial lysates containing the scFv (1:10 dilution) were used as primary binding reagents. Purified scFv antibodies (10 µg/mL) were used for analysis of FN binding and cross-reactivity. Bound scFvs were detected using the same anti-HA and secondary antibody combination. After three washings using PBST, signals were developed using diaminobenzidine (DAB) for Figure 1D, Figure 4A,B, and Figure 6A, or enhanced chemiluminescence (ECL) for Figure 6B, Figure 7B, and Supplementary Figure S1. Blocking, washing, incubation, and detection conditions were identical to those described above.

### 4.7. Enzyme-Linked Immunosorbent Assay (ELISA)

Recombinant FN, BSA, fibrinogen, HSA, or collagens I, III, and IV (0.5 µg/well) were immobilized onto ELISA plates by overnight incubation at 4 °C. Wells were blocked with 5% skim milk in PBS for 1 h at room temperature. For analysis of the rabbit immune response, rabbit sera from pre-immunization and post-fourth immunization were serially diluted (1:1000–1:128,000) and incubated in FN- or BSA-coated wells at 37 °C for 1 h. After six washes with PBST, bound antibodies were detected with HRP-conjugated goat anti-rabbit IgG for 1 h at room temperature. Following additional washes, reactions were developed with 3,3′,5,5′-tetramethylbenzidine (TMB; iReal Biotechnology Inc., Hsinchu, Taiwan) and stopped with 1 N HCl. Optical density (OD) was measured at 450 nm.

For phage-based ELISA, amplified phages (10^11^–10^12^ PFU) from each round of biopanning were added to FN-coated wells and detected with HRP-conjugated mouse anti-M13 antibody (Sino Biological Inc., Beijing, China). To further confirm binding activity, scFv antibodies expressed in *E. coli* lysates (1:10 dilution) were analyzed using rabbit anti-HA antibody, followed by HRP-conjugated goat anti-rabbit IgG. For ELISA titration of the purified scFv antibodies, the proteins were serially diluted two-fold from 20 to 0.01 μg/mL and added to FN- or BSA-coated wells. For cross-reactivity analysis, purified scFvs were tested at 10 μg/mL against FN, fibrinogen, HSA, collagens I, III, and IV, and BSA. Post-immunization rabbit serum diluted 1:10,000 was analyzed against the same protein panel. Bound purified scFvs were detected using rabbit anti-HA antibody followed by HRP-conjugated goat anti-rabbit IgG, whereas bound rabbit serum antibodies were detected directly using HRP-conjugated goat anti-rabbit IgG. All blocking, washing, incubation, and detection steps were performed as described above. ELISA results are presented as the mean ± standard deviation (SD) of duplicate measurements.

### 4.8. Sequence Analysis of Selected scFv Antibodies

Nucleotide sequences of randomly selected scFv clones were determined using the primer ompseq (5′-AAGACAGCTATCGCGATTGCAGTG-3′). The deduced amino acid sequences of V_L_ and V_H_ regions, including framework (FR) and complementarity-determining regions (CDRs), were aligned with rabbit immunoglobulin germline genes using the BioEdit program (version 7.2.5) [42].

### 4.9. FOPPR-Based Kinetic and Sequential-Binding Competition Analyses

The binding kinetics of the purified fibronectin-binding scFvs were analyzed by Instant NanoBiosensors Co., Ltd. (Taipei, Taiwan) as a fee-for-service analysis using a fiber-optic particle plasmon resonance (FOPPR) biosensor [37]. Following activation of the sensor surface, recombinant human fibronectin prepared at 20 μg/mL was immobilized according to the service provider’s standard procedure. The sensor was subsequently subjected to post-immobilization treatment and PBS washing cycles until a stable baseline was established. Each purified scFv was analyzed at six concentrations prepared by twofold serial dilution: 10, 5, 2.5, 1.25, 0.625, and 0.3125 μg/mL. Concentration-dependent response data from all six concentrations were analyzed using the instrument’s kinetic-analysis software to determine the association rate constant (*ka*) and dissociation rate constant (*kd*). The apparent equilibrium dissociation constant (*K_D_*_,app_) was calculated as *k_d_*/*k_a_*.

For the preliminary sequential-binding competition analysis, recombinant human fibronectin was immobilized onto two FOPPR sensor chips (Instant NanoBiosensors Co., Ltd., Taipei, Taiwan) using a 1 μg/mL coating solution. On each sensor chip, FN-L13 was sequentially introduced until no further detectable decrease in the sensor response was observed, indicating that the binding response had reached a plateau. FN-S1, FN-S4, and FN-S6 were then sequentially introduced onto one FN-L13-prebound sensor surface, whereas FN-S8, FN-L1, and FN-L5 were sequentially introduced onto the second FN-L13-prebound sensor surface. Changes in the FOPPR sensor response were monitored following each scFv addition. The absence of a further detectable decrease in sensor response after addition of a subsequent scFv was interpreted as apparent competition with FN-L13 under the assay conditions [39].

### 4.10. Size-Exclusion Chromatography (SEC) Analysis of Purified scFv Antibodies

The oligomeric states of the purified scFv antibodies were evaluated by analytical size-exclusion chromatography (SEC) using a Waters Alliance e2695 Separations Module (Waters Corporation, Milford, MA, USA) equipped with a Waters 2998 Photodiode Array Detector (Waters Corporation, Milford, MA, USA) and a Phenomenex bioZen dSEC-2 column (Phenomenex, Torrance, CA, USA; 1.8 µm, 200 Å, 300 × 4.6 mm [length × internal diameter]). PBS was used as the mobile phase under isocratic conditions at a flow rate of 0.2 mL/min, and protein elution was monitored at 280 nm. Bovine serum albumin (BSA; approximately 66 kDa) was analyzed under identical chromatographic conditions as a molecular-size reference. The seven purified recombinant scFv clones, FN-S1, FN-S4, FN-S6, FN-S8, FN-L1, FN-L5, and FN-L13, were analyzed using the same SEC conditions.

## Figures and Tables

**Table 1:** Classification of randomly selected scFv antibodies based on V_L_ and V_H_ sequence identity.

Name	Short-Linker Clones			Name	Long-Linker Clones		
	Light Chain (V_L_)	Heavy Chain (V_H_)	Percentage (%)		Light Chain (V_L_)	Heavy Chain (V_H_)	Percentage (%)
FN-S1	1, 2, 5, 7, 11	1, 2, 5, 7, 11	5/13; 38.5%	FN-L1	1, 2, 4, 6, 9, 10, 12	1, 2, 4, 6, 9, 10, 12	7/13; 53.8%
FN-S3	3	3	1/13; 7.7%	FN-L3	3	3	1/13; 7.7%
FN-S4	4, 9, 12	4, 9, 12	3/13; 23.1%	FN-L5	5, 7, 8, 11	5, 7, 8, 11	4/13; 30.8%
FN-S6	6, 10	6, 10	2/13; 15.4%	FN-L13	13	13	1/13; 7.7%
FN-S8	8, 13	8, 13	2/13; 15.4%				

**Table 2:** Amino acid divergence from the assigned germline sequences in the V_L_ and V_H_ regions of selected rabbit-derived scFv antibodies.

Region	CDR1	CDR2	CDR3	Total CDRs	FR1	FR2	FR3	FR4	Total FRs
V_L_	0–50%	0–33%	0–10%	0–25%	4–32%	0–18%	0–24%	0%	2–21%
V_H_	25–50%	57–75%	0–27%	22–37%	0–16%	6–35%	8–32%	0%	4–23%

V_L_, variable light chain; V_H_, variable heavy chain; CDR, complementarity-determining region; FR, framework region.

**Table 3:** Purification yields of selected rabbit-derived FN-binding constructs.

Clone	Linker Format	Purification Yield (mg/L *E. coli* Culture)
FN-S1	Short	0.56
FN-S4	Short	0.31
FN-S6	Short	0.30
FN-S8	Short	0.25
FN-L1	Long	0.81
FN-L5	Long	0.60
FN-L13	Long	0.80

**Table 4:** Binding kinetic parameters of the purified FN-binding scFvs determined by FOPPR.

Clone	*k_a_* (M^−1^s^−1^)	*k_d_* (s^−1^)	*K_D,app_* (nM)
FN-S1	1.377 × 10^5^	1.393 × 10^−2^	101.17
FN-S4	4.549 × 10^4^	7.028 × 10^−4^	15.45
FN-S6	6.947 × 10^4^	7.121 × 10^−3^	102.51
FN-S8	3.389 × 10^4^	1.464 × 10^−2^	432.06
FN-L1	4.109 × 10^4^	1.474 × 10^−2^	358.71
FN-L5	9.752 × 10^4^	1.915 × 10^−2^	196.34
FN-L13	4.858 × 10^4^	5.445 × 10^−4^	11.21

*k_a_*: association rate constant; *k_d_*: dissociation rate constant; *K_D,app_*: apparent equilibrium dissociation constant.

## Data Availability

The original contributions presented in this study are included in the article/Supplementary Material. Further inquiries can be directed to the corresponding authors.

## Supplementary Materials

The following are available online at https://www.mdpi.com/article/10.3390/ijms27209074/s1.
