# Supplementary material for: Construction and Characterization of Rabbit scFv Antibodies Targeting Fibronectin by Phage Display Technology
Source: Int J Mol Sci. 2026 Oct 13;27(20):9074. doi: 10.3390/ijms27209074 (PMC13705019; doi:10.3390/ijms27209074)
Supplement: Supplementary file 1 [file ijms-27-09074-s001.zip › FOPPR 7 FN-scFv/FN-L1 FOPPR.pdf]

INB-D200 Report

Date / Time : 20260716

Project Name : 5

Sensorgram

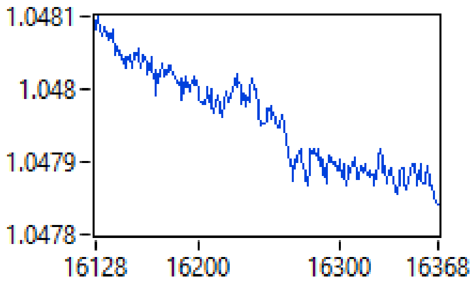

Sample Concentration (ng/ml)	Signal ratio	RMS Start Time (sec)	RMS Stop Time (sec)
0.00	NaN	9183	9282
0.00	NaN	-99	0
312.50	0.002630	10171	10270
625.00	0.006085	11025	11124
1250.00	0.012836	12191	12290
2500.00	0.023950	13583	13682
5000.00	0.043822	14976	15075
10000.00	0.072594	16210	16309

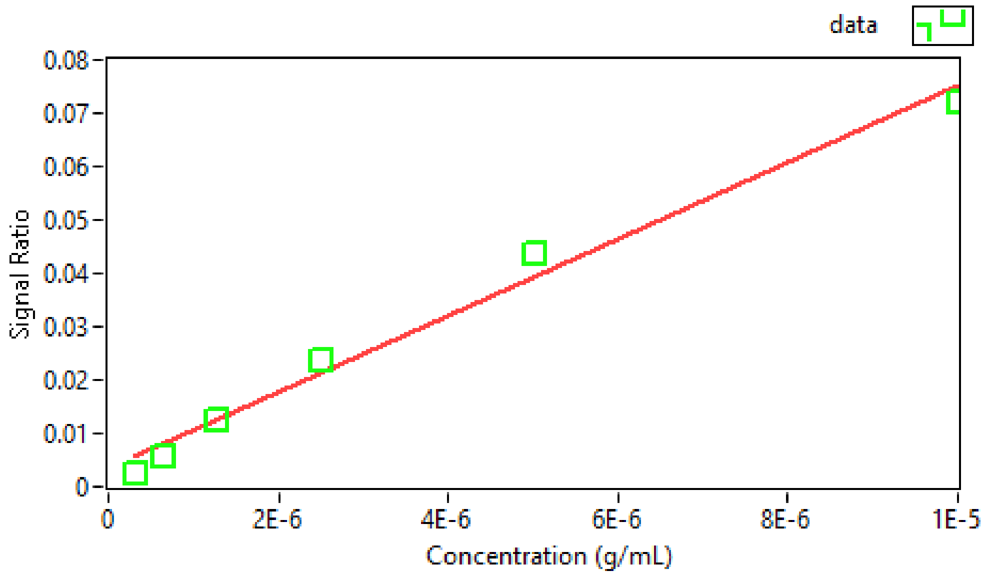

Sample name	5
Sample M.W. (kDa)	0.00

Equation	Y = a + bx
Slope	7.1951E+3
Intercept	3.3773E-3
Buffer SD	2.5117E-5
R-square	0.9871

LOD (ng/mL)	1.0472E+1
LOQ (ng/mL)	3.4908E+1

INB-D200 Binding Kinetic Analysis Report

Date / Time : 20260716

Project Name :

Kinetic Analysis

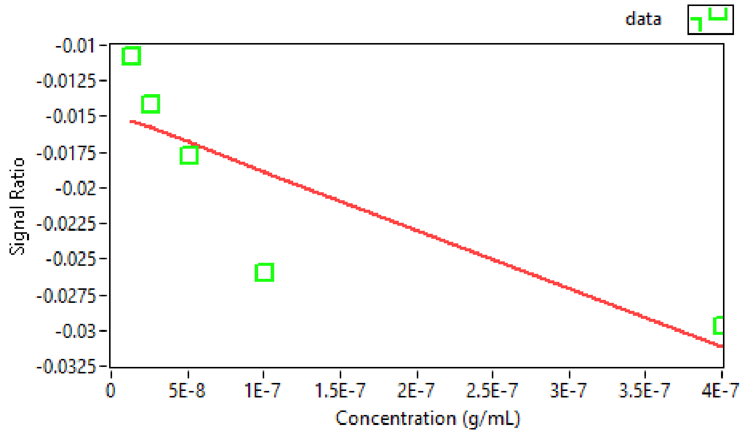

Association Rate Constant (ka) : 4.109E+4 (1/Ms)

Dissociation Rate Constant (kd) : 1.474E-2 (1/s)

Affinity Constant (KD): 358.71 (nM)

Coefficient of Determination (R-square): 0.6959
