# Supplementary material for: Construction and Characterization of Rabbit scFv Antibodies Targeting Fibronectin by Phage Display Technology
Source: Int J Mol Sci. 2026 Oct 13;27(20):9074. doi: 10.3390/ijms27209074 (PMC13705019; doi:10.3390/ijms27209074)
Supplement: Supplementary file 1 [file ijms-27-09074-s001.zip › FOPPR 7 FN-scFv/FN-L13 FOPPR.pdf]

INB-D200 Report

Date / Time : 20260716

Project Name : 7

Sensorgram

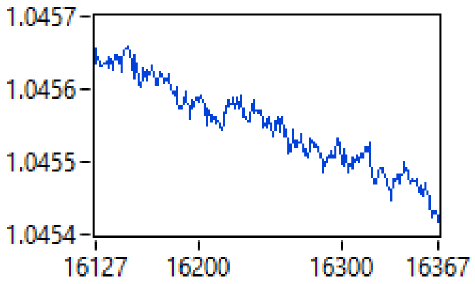

Sample Concentration (ng/ml)	Signal ratio	RMS Start Time (sec)	RMS Stop Time (sec)
0.00	NaN	9204	9303
0.00	NaN	-99	0
312.50	-0.000631	10182	10281
625.00	-0.000317	11038	11137
1250.00	0.001091	12202	12301
2500.00	0.003630	13594	13693
5000.00	0.009896	14986	15085
10000.00	0.018577	16248	16347

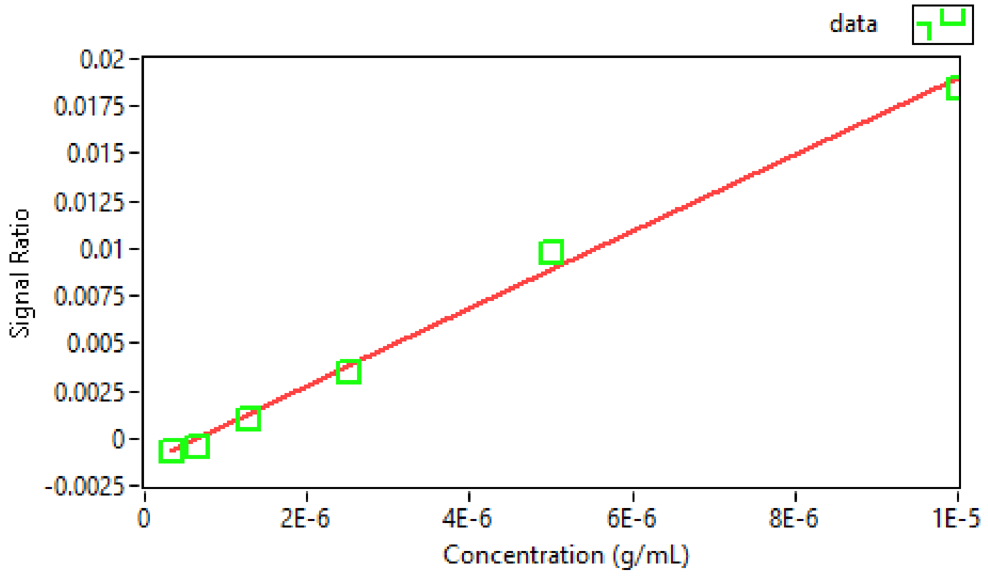

Sample name	7
Sample M.W. (kDa)	0.00

Equation	Y = a + bx
Slope	2.0304E+3
Intercept	-1.2877E-3
Buffer SD	1.3921E-5
R-square	0.9951

LOD (ng/mL)	2.0569E+1
LOQ (ng/mL)	6.8562E+1

INB-D200 Binding Kinetic Analysis Report

Date / Time : 20260716

Project Name :

Kinetic Analysis

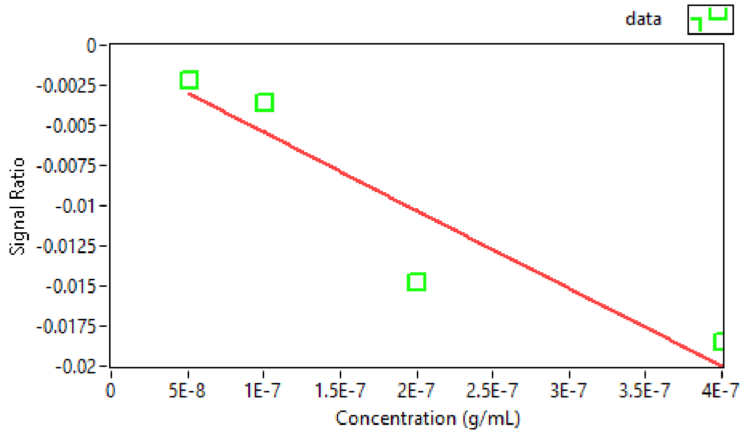

Association Rate Constant (ka) : 4.858E+4 (1/Ms)

Dissociation Rate Constant (kd) : 5.445E-4 (1/s)

Affinity Constant (KD): 11.21 (nM)

Coefficient of Determination (R-square): 0.8650
