# Supplementary material for: Construction and Characterization of Rabbit scFv Antibodies Targeting Fibronectin by Phage Display Technology
Source: Int J Mol Sci. 2026 Oct 13;27(20):9074. doi: 10.3390/ijms27209074 (PMC13705019; doi:10.3390/ijms27209074)
Supplement: Supplementary file 1 [file ijms-27-09074-s001.zip › FOPPR 7 FN-scFv/FN-L5 FOPPR.pdf]

INB-D200 Report

Date / Time : 20260716

Project Name : 6

Sensorgram

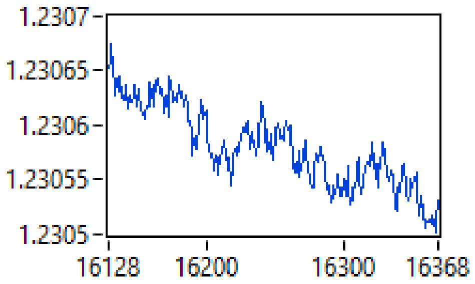

Sample Concentration (ng/ml)	Signal ratio	RMS Start Time (sec)	RMS Stop Time (sec)
0.00	NaN	9199	9298
0.00	NaN	-99	0
312.50	0.000812	10177	10276
625.00	0.001633	11031	11130
1250.00	0.003299	12196	12295
2500.00	0.006939	13589	13688
5000.00	0.013692	14981	15080
10000.00	0.026380	16243	16342

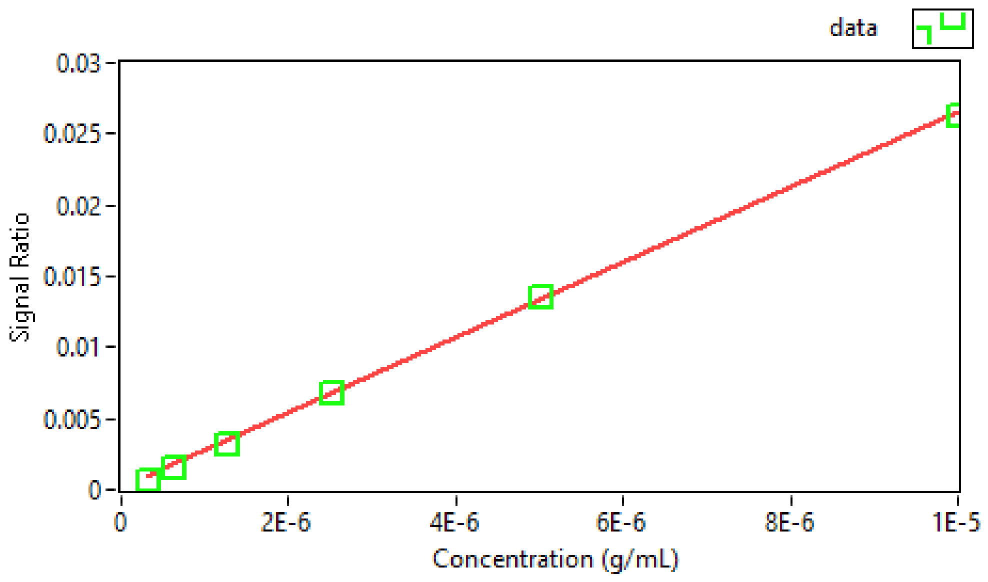

Sample name	6
Sample M.W. (kDa)	0.00

Equation	Y = a + bx
Slope	2.6478E+3
Intercept	1.0445E-4
Buffer SD	1.4759E-5
R-square	0.9995

LOD (ng/mL)	1.6722E+1
LOQ (ng/mL)	5.5741E+1

INB-D200 Binding Kinetic Analysis Report

Date / Time : 20260716

Project Name :

Kinetic Analysis

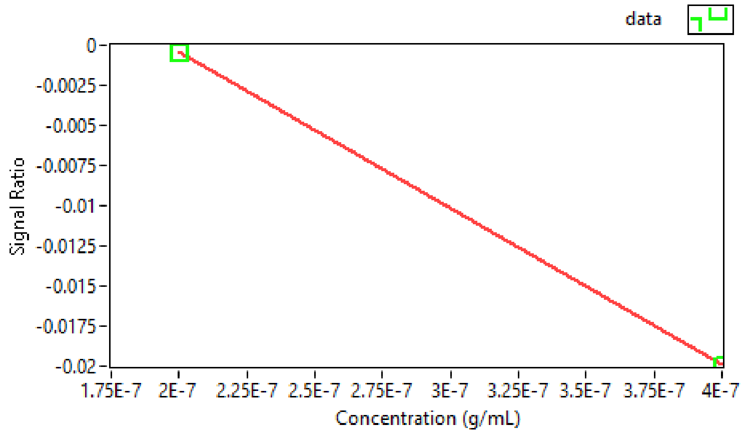

Association Rate Constant (ka) : 9.752E+4 (1/Ms)

Dissociation Rate Constant (kd) : 1.915E-2 (1/s)

Affinity Constant (KD): 196.34 (nM)

Coefficient of Determination (R-square): 1.0000
