# Supplementary material for: Construction and Characterization of Rabbit scFv Antibodies Targeting Fibronectin by Phage Display Technology
Source: Int J Mol Sci. 2026 Oct 13;27(20):9074. doi: 10.3390/ijms27209074 (PMC13705019; doi:10.3390/ijms27209074)
Supplement: Supplementary file 1 [file ijms-27-09074-s001.zip › FOPPR 7 FN-scFv/FN-S1 FOPPR.pdf]

INB-D200 Report

Date / Time : 20260716

Project Name : 1

Sensorgram

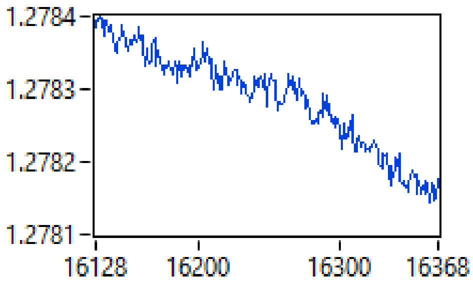

Sample Concentration (ng/ml)	Signal ratio	RMS Start Time (sec)	RMS Stop Time (sec)
0.00	NaN	9154	9253
0.00	NaN	-99	0
312.50	0.001982	10151	10250
625.00	0.004523	10999	11098
1250.00	0.009565	12164	12263
2500.00	0.019220	13561	13660
5000.00	0.036420	14954	15053
10000.00	0.064009	16194	16293

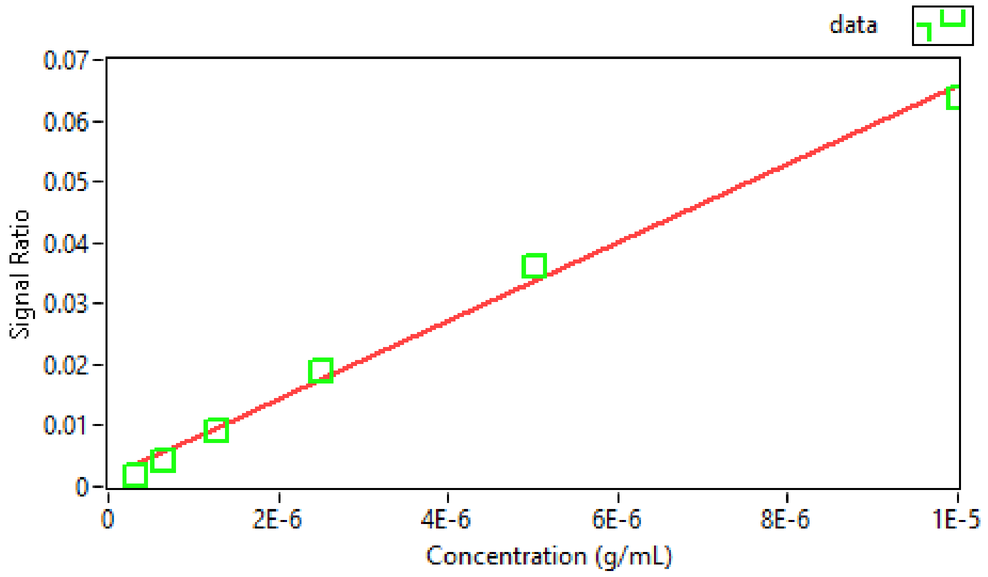

Sample name	1
Sample M.W. (kDa)	0.00

Equation	Y = a + bx
Slope	6.4091E+3
Intercept	1.5899E-3
Buffer SD	3.2257E-5
R-square	0.9941

LOD (ng/mL)	1.5099E+1
LOQ (ng/mL)	5.0330E+1

INB-D200 Binding Kinetic Analysis Report

Date / Time : 20260716

Project Name :

Kinetic Analysis

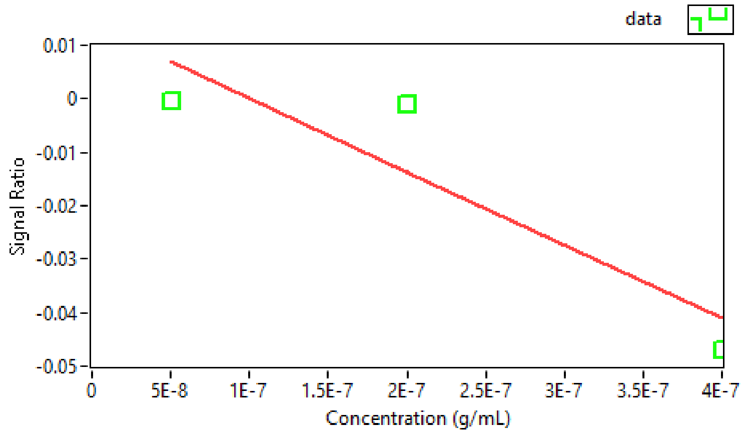

Association Rate Constant (ka) : 1.377E+5 (1/Ms)

Dissociation Rate Constant (kd) : 1.393E-2 (1/s)

Affinity Constant (KD): 101.17 (nM)

Coefficient of Determination (R-square): 0.8241
