# Supplementary material for: Construction and Characterization of Rabbit scFv Antibodies Targeting Fibronectin by Phage Display Technology
Source: Int J Mol Sci. 2026 Oct 13;27(20):9074. doi: 10.3390/ijms27209074 (PMC13705019; doi:10.3390/ijms27209074)
Supplement: Supplementary file 1 [file ijms-27-09074-s001.zip › FOPPR 7 FN-scFv/FN-S4 FOPPR.pdf]

INB-D200 Report

Date / Time : 20260716

Project Name : 2

Sensorgram

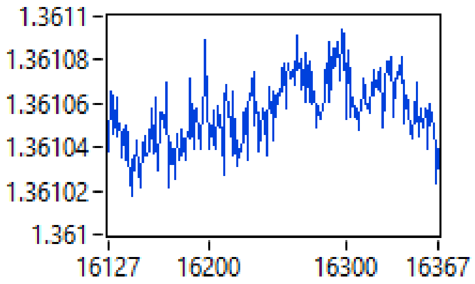

Sample Concentration (ng/ml)	Signal ratio	RMS Start Time (sec)	RMS Stop Time (sec)
0.00	NaN	9162	9261
0.00	NaN	-99	0
312.50	-0.000364	10156	10255
625.00	-0.000334	11004	11103
1250.00	-0.000270	12171	12270
2500.00	0.000132	13566	13665
5000.00	0.001425	14961	15060
10000.00	0.003843	16198	16297

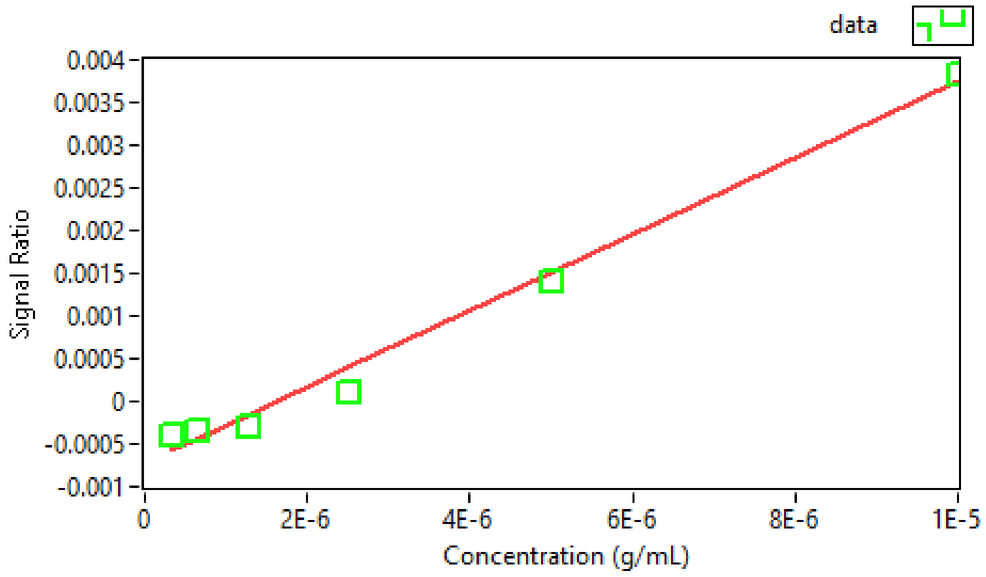

Sample name	2
Sample M.W. (kDa)	0.00

Equation	Y = a + bx
Slope	4.4661E+2
Intercept	-7.2679E-4
Buffer SD	3.1675E-5
R-square	0.9887

LOD (ng/mL)	2.1277E+2
LOQ (ng/mL)	7.0923E+2

INB-D200 Binding Kinetic Analysis Report

Date / Time : 20260716

Project Name :

Kinetic Analysis

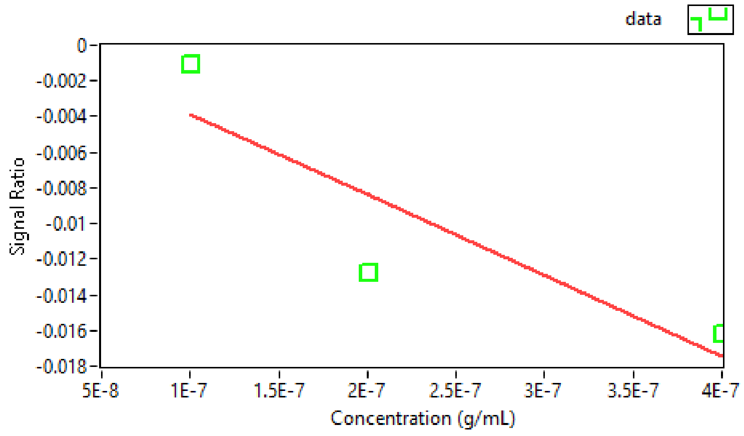

Association Rate Constant (ka) : 4.549E+4 (1/Ms)

Dissociation Rate Constant (kd) : 7.028E-4 (1/s)

Affinity Constant (KD): 15.45 (nM)

Coefficient of Determination (R-square): 0.7739
