# Supplementary material for: Construction and Characterization of Rabbit scFv Antibodies Targeting Fibronectin by Phage Display Technology
Source: Int J Mol Sci. 2026 Oct 13;27(20):9074. doi: 10.3390/ijms27209074 (PMC13705019; doi:10.3390/ijms27209074)
Supplement: Supplementary file 1 [file ijms-27-09074-s001.zip › FOPPR 7 FN-scFv/FN-S6 FOPPR.pdf]

INB-D200 Report

Date / Time : 20260716

Project Name : 3

Sensorgram

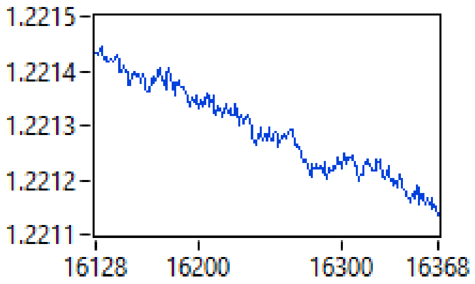

Sample Concentration (ng/ml)	Signal ratio	RMS Start Time (sec)	RMS Stop Time (sec)
0.00	NaN	9167	9266
0.00	NaN	-99	0
312.50	0.001345	10161	10260
625.00	0.003171	11010	11109
1250.00	0.006893	12178	12277
2500.00	0.013531	13572	13671
5000.00	0.023774	14965	15064
10000.00	0.041427	16203	16302

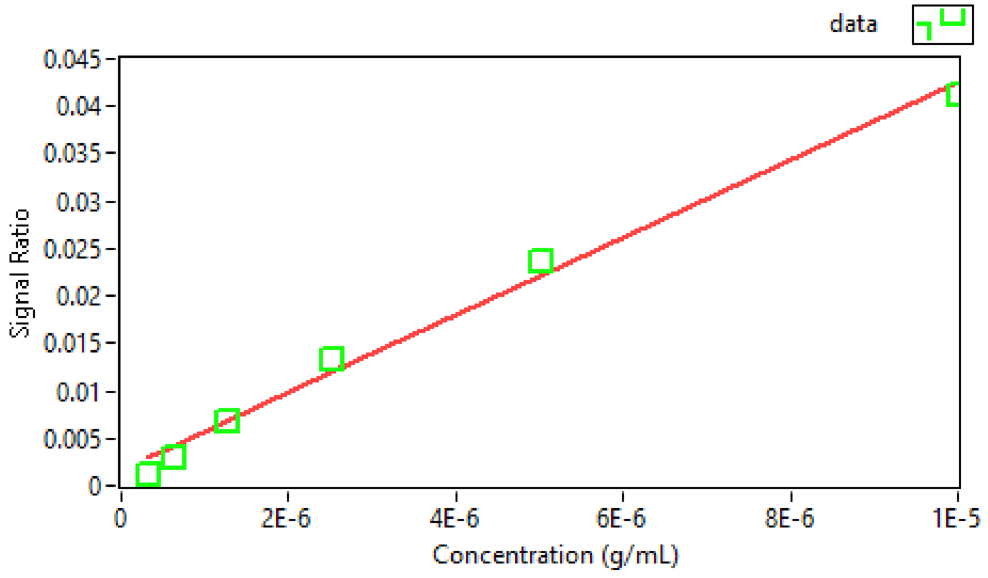

Sample name	3
Sample M.W. (kDa)	0.00

Equation	Y = a + bx
Slope	4.1078E+3
Intercept	1.5447E-3
Buffer SD	1.2634E-5
R-square	0.9911

LOD (ng/mL)	9.2265E+0
LOQ (ng/mL)	3.0755E+1

INB-D200 Binding Kinetic Analysis Report

Date / Time : 20260716

Project Name :

Kinetic Analysis

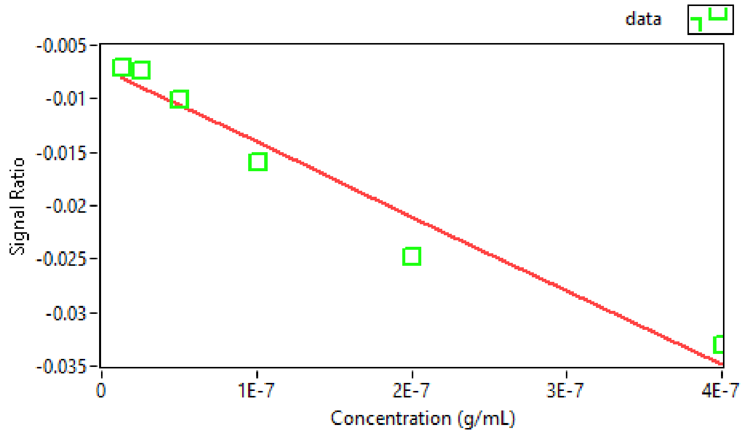

Association Rate Constant (ka) : 6.947E+4 (1/Ms)

Dissociation Rate Constant (kd) : 7.121E-3 (1/s)

Affinity Constant (KD): 102.51 (nM)

Coefficient of Determination (R-square): 0.9570
