# Supplementary material for: Construction and Characterization of Rabbit scFv Antibodies Targeting Fibronectin by Phage Display Technology
Source: Int J Mol Sci. 2026 Oct 13;27(20):9074. doi: 10.3390/ijms27209074 (PMC13705019; doi:10.3390/ijms27209074)
Supplement: Supplementary file 1 [file ijms-27-09074-s001.zip › FOPPR 7 FN-scFv/FN-S8 FOPPR.pdf]

INB-D200 Report

Date / Time : 20260716

Project Name : 4

Sensorgram

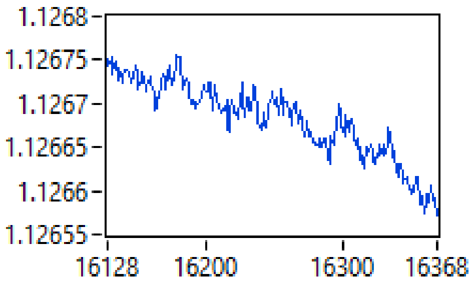

Sample Concentration (ng/ml)	Signal ratio	RMS Start Time (sec)	RMS Stop Time (sec)
0.00	NaN	9173	9272
0.00	NaN	-99	0
312.50	0.002067	10166	10265
625.00	0.004766	11020	11119
1250.00	0.009709	12184	12283
2500.00	0.020954	13577	13676
5000.00	0.039402	14970	15069
10000.00	0.067234	16220	16319

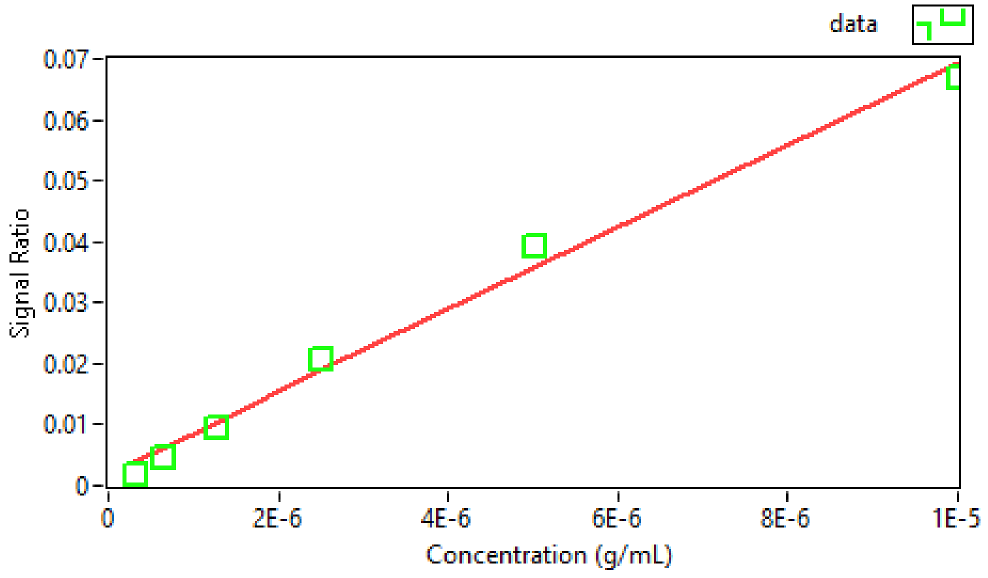

Sample name	4
Sample M.W. (kDa)	0.00

Equation	Y = a + bx
Slope	6.7621E+3
Intercept	1.8338E-3
Buffer SD	1.4864E-5
R-square	0.9907

LOD (ng/mL)	6.5943E+0
LOQ (ng/mL)	2.1981E+1

INB-D200 Binding Kinetic Analysis Report

Date / Time : 20260716

Project Name :

Kinetic Analysis

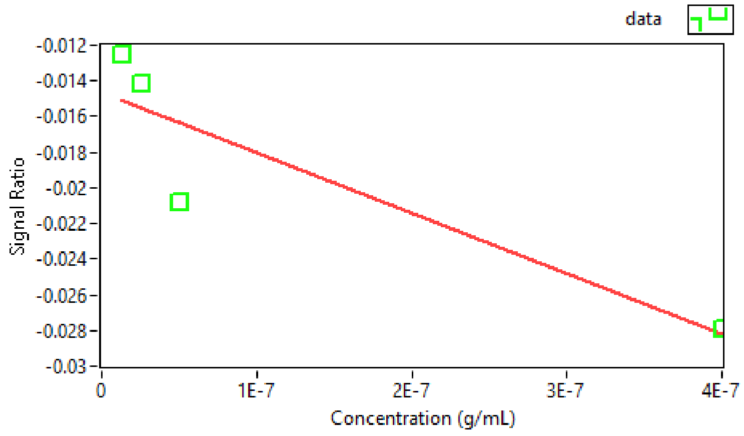

Association Rate Constant (ka) : 3.389E+4 (1/Ms)

Dissociation Rate Constant (kd) : 1.464E-2 (1/s)

Affinity Constant (KD): 432.06 (nM)

Coefficient of Determination (R-square): 0.8075
