# Supplementary material for: Construction and Characterization of Rabbit scFv Antibodies Targeting Fibronectin by Phage Display Technology
Source: Int J Mol Sci. 2026 Oct 13;27(20):9074. doi: 10.3390/ijms27209074 (PMC13705019; doi:10.3390/ijms27209074)
Supplement: Supplementary file 1 [file ijms-27-09074-s001.zip › Supplementary Figure S1.pdf]

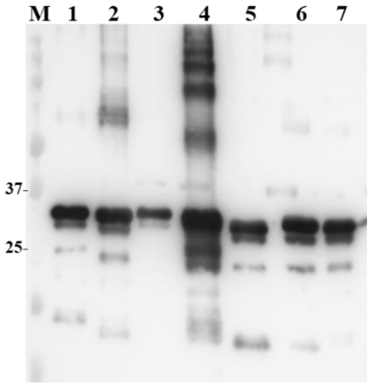

Figure S1. Western blot analysis of purified rabbit scFv antibodies using an anti-HA antibody. Seven purified scFv antibodies were analyzed by Western blotting using an anti-HA antibody recognizing the C-terminal HA tag. All constructs showed major HA-reactive bands within the expected scFv molecular-weight range, with additional HA-reactive bands observed in several preparations. Closely migrating double-band patterns were particularly evident for FN-L1 and FN-L13, indicating that both bands retained the C-terminal HA tag. The precise molecular identities of these additional species were not further determined. Lane 1, FN-S1; lane 2, FN-S4; lane 3, FN-S6; lane 4, FN-S8; lane 5, FN-L1; lane 6, FN-L5; lane 7, FN-L13.
