# Supplementary material for: Construction and Characterization of Rabbit scFv Antibodies Targeting Fibronectin by Phage Display Technology
Source: Int J Mol Sci. 2026 Oct 13;27(20):9074. doi: 10.3390/ijms27209074 (PMC13705019; doi:10.3390/ijms27209074)
Supplement: Supplementary file 1 [file ijms-27-09074-s001.zip › Supplementary Figure S2.pdf]

(A)

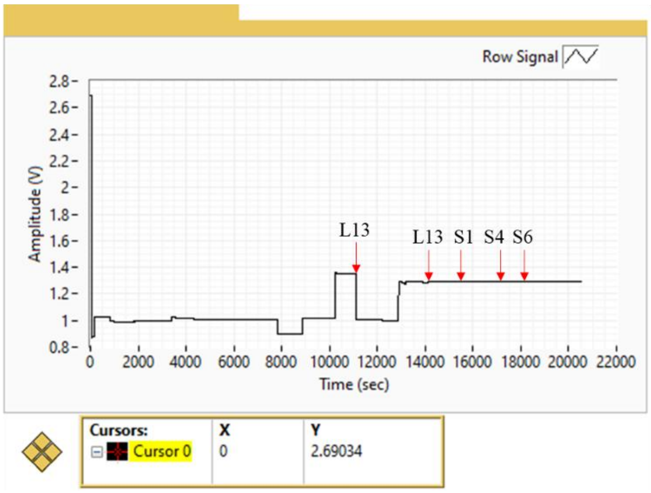

(B)

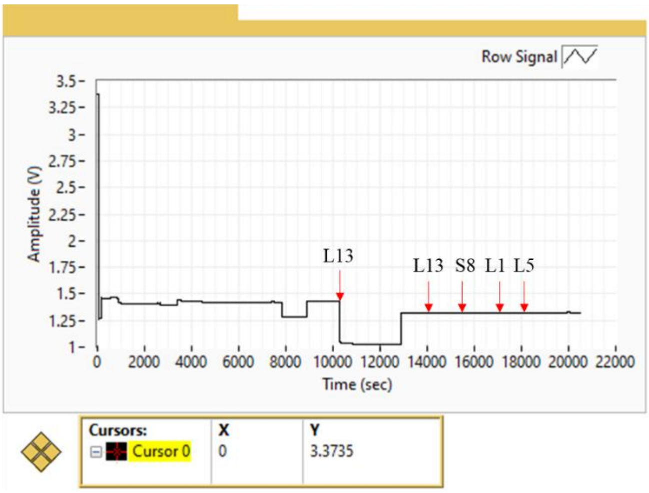

Figure S2. Preliminary FOPPR-based sequential-binding analysis of rabbit scFv antibodies against fibronectin. Recombinant human fibronectin (FN; 1 µg/mL) was immobilized on two FOPPR sensor chips according to the standard procedure of the service provider. On each sensor chip, FN-L13 was sequentially introduced until no further detectable decrease in sensor response was observed. The remaining scFvs were then sequentially introduced after the FN-L13 response had reached a plateau. (A) FN-S1, FN-S4, and FN-S6 were sequentially introduced following FN-L13. (B) FN-S8, FN-L1, and FN-L5 were sequentially introduced following FN-L13. Red arrows indicate the approximate time points at which each scFv was introduced. No further detectable decrease in sensor response was observed following addition of the six tested scFvs, suggesting apparent competition with FN-L13 under the assay conditions.
