# Supplementary material for: Construction and Characterization of Rabbit scFv Antibodies Targeting Fibronectin by Phage Display Technology
Source: Int J Mol Sci. 2026 Oct 13;27(20):9074. doi: 10.3390/ijms27209074 (PMC13705019; doi:10.3390/ijms27209074)
Supplement: Supplementary file 1 [file ijms-27-09074-s001.zip › Supplementary Figure S3.pdf]

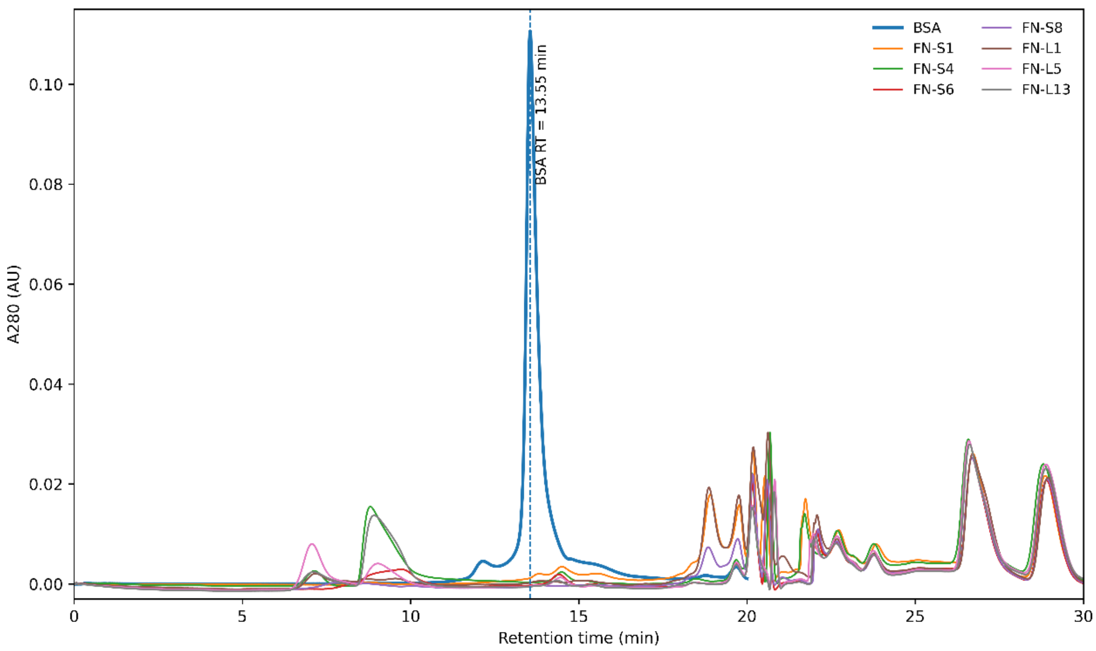

Figure S3. Analytical size-exclusion chromatography (SEC) profiles of purified rabbit scFv antibodies. Seven purified scFv antibodies (FN-S1, FN-S4, FN-S6, FN-S8, FN-L1, FN-L5, and FN-L13) were analyzed by analytical size-exclusion chromatography using a Waters Alliance e2695 Separations Module equipped with a Waters 2998 Photodiode Array Detector and a Phenomenex bioZen dSEC-2 column (1.8 µm, 200 Å, 300 × 4.6 mm). PBS was used as the mobile phase under isocratic conditions at a flow rate of 0.2 mL/min, and protein elution was monitored at 280 nm. Bovine serum albumin (BSA; ~66 kDa) was analyzed under the same conditions as a molecular-size reference and eluted at approximately 13.55 min. The chromatograms were replotted directly from the raw time-absorbance data exported from the Waters system without curve fitting or smoothing.
