# Supplementary material for: Construction and Characterization of Rabbit scFv Antibodies Targeting Fibronectin by Phage Display Technology
Source: Int J Mol Sci. 2026 Oct 13;27(20):9074. doi: 10.3390/ijms27209074 (PMC13705019; doi:10.3390/ijms27209074)
Supplement: Supplementary file 1 [file ijms-27-09074-s001.zip › Supplementary Figure S4.pdf]

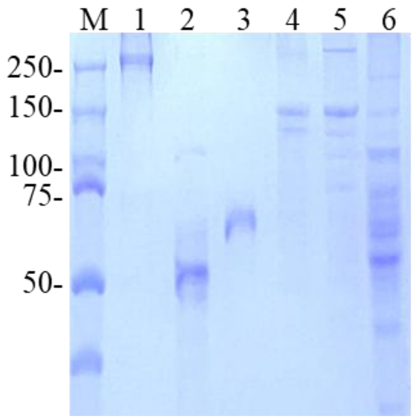

Figure S4. SDS-PAGE profiles of fibronectin and selected ECM-associated and plasma proteins used for binding selectivity and cross-reactivity analysis. Recombinant human fibronectin (FN), fibrinogen, human serum albumin (HSA), and collagens I, III, and IV (0.5 µg per lane) were separated by SDS-PAGE and visualized by Coomassie Brilliant Blue staining. Lane M, molecular weight marker; lane 1, FN; lane 2, fibrinogen; lane 3, HSA; lane 4, collagen I; lane 5, collagen III; lane 6, collagen IV. The collagen preparations displayed heterogeneous banding patterns, including lower-molecular-weight species in the collagen IV preparation.
