# Supplementary material for: Construction and Characterization of Rabbit scFv Antibodies Targeting Fibronectin by Phage Display Technology
Source: Int J Mol Sci. 2026 Oct 13;27(20):9074. doi: 10.3390/ijms27209074 (PMC13705019; doi:10.3390/ijms27209074)
Supplement: Supplementary file 1 [file ijms-27-09074-s001.zip › Supplementary Table S1.pdf]

Table S1. Preliminary FOPPR-based sequential-binding analysis of FN-binding scFvs using FN-L13 as the reference scFv.

FOPPR sensor chip	First-bound scFv	Subsequently injected scFv	Change in FOPPR response	Preliminary interpretation
1	FN-L13	FN-S1	No further detectable decrease	Apparent competition with FN-L13
		FN-S4	No further detectable decrease	Apparent competition with FN-L13
		FN-S6	No further detectable decrease	Apparent competition with FN-L13
2	FN-L13	FN-S8	No further detectable decrease	Apparent competition with FN-L13
		FN-L1	No further detectable decrease	Apparent competition with FN-L13
		FN-L5	No further detectable decrease	Apparent competition with FN-L13

FN-L13 was sequentially added to the FN-coated sensor until the response reached a plateau. The test scFvs are listed in the order of sequential addition on each sensor chip. The absence of a further detectable decrease following addition of a subsequent scFv was interpreted as apparent competition with FN-L13 under the assay conditions.
